# Genomic Insights into Basal Diptera Phylogeny: The Non-Monophyletic Nature of Blephariceromorpha

**DOI:** 10.3390/ijms26125714

**Published:** 2025-06-14

**Authors:** Yaoming Yang, Jiayao Ren, Xuhongyi Zheng, Lingna Cai, Jiayin Guan, Tianlong Cai, Xiaodong Xu, Ying Zhen

**Affiliations:** 1 College of Life Sciences, Zhejiang University, Hangzhou 310058, China; yangyaoming@westlake.edu.cn; 2 Research Center for Industries of the Future, School of Life Sciences, Westlake University, Hangzhou 310030, China; 3 Westlake Laboratory of Life Sciences and Biomedicine, Hangzhou 310030, China; 4 College of Plant Protection, Anhui Agricultural University, Hefei 230036, China; 5 The Key Laboratory of Jiangsu Biodiversity and Biotechnology, College of Life Sciences, Nanjing Normal University, Nanjing 210023, China; 6 Institute of Biology, Westlake Institute for Advanced Study, Hangzhou 310024, China; 7 School of Life Sciences, Fudan University, Shanghai 200433, China

**Keywords:** Diptera, mitogenomes, Nematocera, nuclear orthologs, phylogenomics

## Abstract

Diptera is one of the most ecologically significant and species-rich insect orders, but there are still unresolved phylogenetic relationships among its basal lineages, particularly within the infraorder Blephariceromorpha, due to limited molecular data. To address this gap, this study employs two parallel genomic approaches: mitochondrial genomes and nuclear genomic analysis, covering 64 families and over 100 species of Diptera and their outgroups, to elucidate these phylogenetic relationships. Our results strongly support the monophyly of each constituent family (Blephariceridae, Deuterophlebiidae, and Nymphomyiidae), yet they reject the monophyly of Blephariceromorpha. Crucially, we found that Deuterophlebiidae and Nymphomyiidae form a sister group representing the basal-most lineage of Diptera, whereas Blephariceridae is positioned within Psychodomorpha. This indicates that the similar larval habitats and morphological traits shared between Blephariceridae and the Nymphomyiidae + Deuterophlebiidae clade are the result of convergent evolution. By resolving long-standing debates on the relationships within Blephariceromorpha and the basal lineages of Diptera, this study provides new insights into the evolutionary history of Diptera, especially within the suborder Nematocera.

## 1. Introduction

Diptera, commonly known as true flies, represent one of the largest insect orders, comprising over 165,000 described species [1]. This order traditionally is classified into two primary suborders, the paraphyletic Nematocera (meaning “thread-horns”) includes all flies except those from the monophyly suborder Brachycera (meaning “short-horns”), which encompasses more familiar species such as the housefly and the common fruit fly [2,3,4,5,6]. Nematocera, encompassing approximately 52,000 species distributed across 36 families, is characterized by elongated bodies and long, multi-segmented antennae [1,7]. Species within this suborder exhibit remarkable morphological and ecological diversity, with species occupying virtually every terrestrial and aquatic habitat. Their feeding strategies range from hematophagy, predation, and parasitism to herbivory and saprophagy [8]. Understanding the phylogeny of Nematocera is crucial for elucidating their evolutionary history, interpreting their ecological functions from decomposition to pollination, and assessing their socioeconomic impact, ranging from mosquito-borne diseases to agricultural pest management [9,10,11,12].

Within the systematic classification of Nematocera, the infraorder Blephariceromorpha (comprising Blephariceridae, Deuterophlebiidae, and Nymphomyiidae) has remained one of the most contentious issues in dipteran systematics [2,3,4,5,7,13,14,15,16,17,18,19]. Traditional morphological analyses grouped these families based on shared larval adaptations to fast-flowing streams (e.g., prolegs and suction organs) [13,15,16]. However, conflicting hypotheses have emerged: (1) Nymphomyiidae as sister to Culicomorpha [20], (2) Deuterophlebiidae and/or Nymphomyiidae as the basal dipteran lineage [2,5,14,17], and (3) Blephariceridae as member of Psychodomorpha [2,5,14]. The unstable position of Deuterophlebiidae (alternatively sister to Blephariceridae or Nymphomyiidae) with conflicting phylogenetic evidence exemplifies how specialized habitat adaptations can obscure phylogenetic signals [2,5,7]. Resolving these conflicts requires nuclear genomic-scale data to overcome limitations of fragmentary molecular datasets and morphological homoplasy.

Recent phylogenetic studies utilize diverse molecular datasets, including mitochondrial genomes and nuclear data, to explore evolutionary relationships [21,22,23,24]. Advances in sequencing technology and analytical methods have significantly expanded the availability of nuclear genomic data, yielding extensive collections of phylogenetically informative orthologous loci that enables the resolution of complex evolutionary relationships across wider taxonomic spectra [21,22,24]. The integrated analysis of mitochondrial and nuclear datasets (both transcriptomic and genomic) offers complementary perspectives for resolving deep evolutionary relationships and establishing robust phylogenetic frameworks for insects [25,26,27]. However, data for Blephariceromorpha remain limited, with only one or two species per family represented by mitochondrial or transcriptomic data [14]. This limitation emphasizes the need to supplement existing datasets to robustly investigate the phylogeny of these groups using both mitochondrial and transcriptomic data.

In this study, we present a comprehensive molecular dataset comprising 11 species from the three families within the infraorder Blephariceromorpha (5 Blephariceridae, 5 Deuterophlebiidae, and 1 Nymphomyiidae), with mitochondrial genomes sequenced for all species and transcriptome data obtained for 8 species (Table 1). To address the phylogenetic controversies within Blephariceromorpha, we implemented complementary mitochondrial and nuclear genomic approaches. Our analyses conclusively demonstrate monophyly for each constituent family (Blephariceridae, Deuterophlebiidae, and Nymphomyiidae) but reject the traditional grouping of Blephariceromorpha. Instead, Deuterophlebiidae and Nymphomyiidae form a sister group as the basal lineage of Diptera, while Blephariceridae is placed within Psychodomorpha. These findings enhance our understanding of Nematocera phylogeny and evolutionary diversification within Diptera.

## 2. Results

### 2.1. Mitogenomic and Transcriptomic Data Analysis

**Figure 1:**
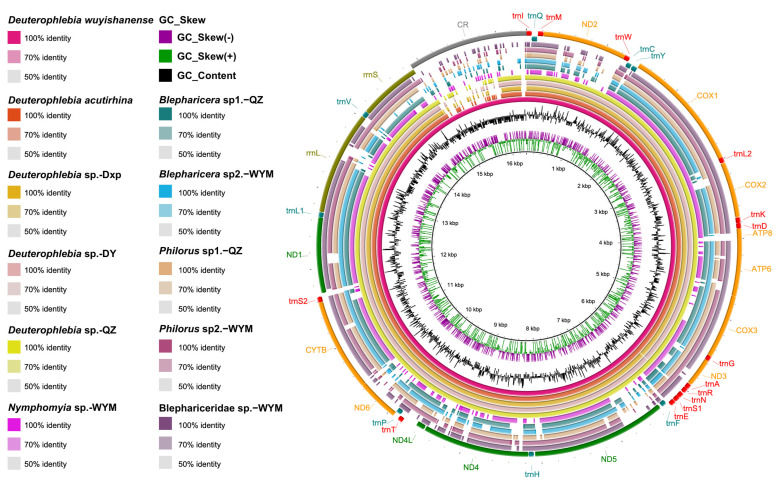
Circular mitogenomes and gene organization of Blephariceridae, Deuterophlebiidae, and Nymphomyiidae. Genes transcribed from the heavy strand are depicted in the outer circle, while those from the light strand are shown in the inner circle.

Our study generated complete or nearly complete mitochondrial genomes of 11 Blephariceromorpha species, including 5 from Blephariceridae, 5 from Deuterophlebiidae, and 1 from Nymphomyiidae, with genome sizes ranging from 16,402 to 16,757 base pairs (bp) (Figure 1 and Table S1). All mitogenomes contained the standard set of 13 protein-coding genes (PCGs), two ribosomal RNA (rRNA) genes, 22 transfer RNA (tRNA) genes, and one control region (CR) (Supplementary Files). Gene arrangement was highly conserved across Diptera. The relative synonymous codon usage (RSCU) varied among families (Figure S1).

**Figure 2:**
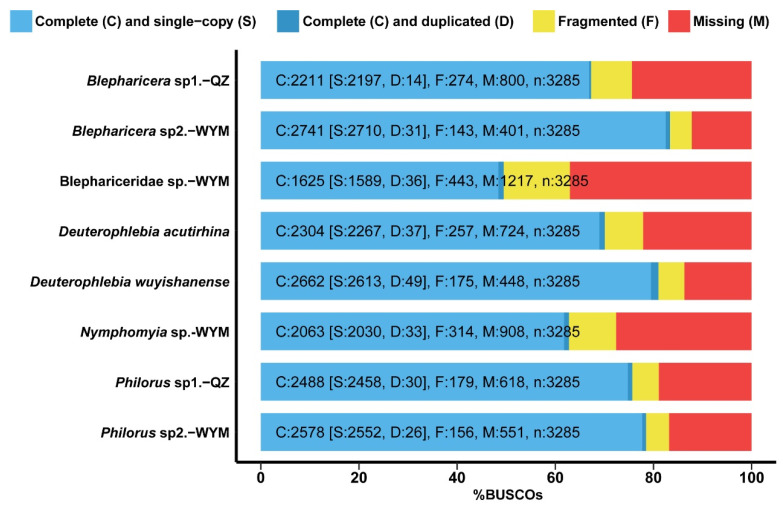
Transcriptome completeness of Blephariceridae, Deuterophlebiidae, and Nymphomyiidae evaluated using BUSCO analysis.

Transcriptomes from eight species were assessed for completeness using BUSCO and assembly metrics (Figure 2, Table S2). Most species demonstrate reliable assembly statistics, with the number of assembled transcripts ranging from 25,847 in *Blepharicera* sp1.-QZ to 53,632 in Blephariceridae sp1.-WYM. The GC content varies from 29.86% in *Deuterophlebia wuyishanense* to 41.39% in Blephariceridae sp1.-WYM. The BUSCO assessments confirmed high assembly quality, with most species containing over 2000 single-copy orthologs, except for Blephariceridae sp1.-WYM (Figure 2, Table S2).

### 2.2. Phylogenetic Analyses Using Nuclear Datasets

**Figure 3:**
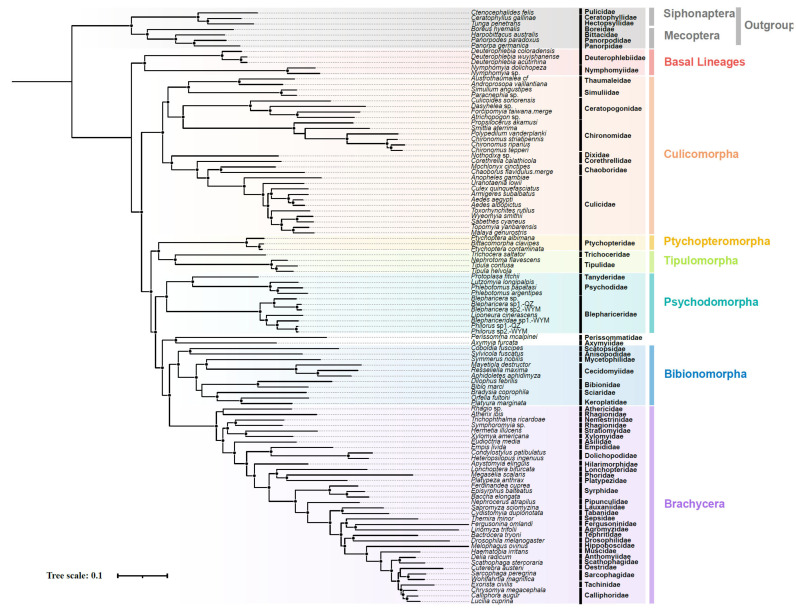
Consensus phylogenetic tree of Diptera based on nuclear USCO amino acids (50–90% taxon occupancy matrices), inferred under maximum likelihood using IQ-TREE (v2.3.4) with GHOST and partitioning models. Circles indicate nodes with ≥98% support (SH-aLRT/UFboot) based on 80% taxon occupancy using partitioning models.

**Figure 4:**
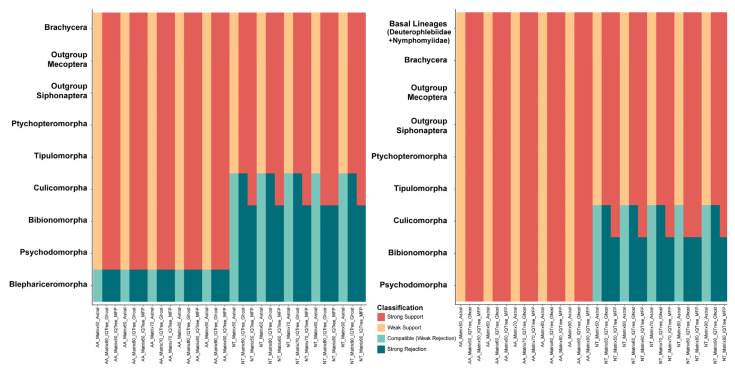
Comparison of nuclear gene phylogenetic inference across different models and data types using DiscoVista (v1.0) analysis. Rows represent major lineages, and columns display results from different modeling schemes and data matrices. “AA” and “NT” represent amino acid and nucleotide data, respectively. Each matrix represents a portion of species included in the analysis, with different methods applied, including IQ-TREE (v2.3.4) ModelFinder Plus (MFP), the Ghost Model, and Astral for species tree inference.

Our phylogenetic reconstruction using nuclear genomic datasets with a varying number of loci and site occupancy levels for both amino acids and nucleotides consistently rejected the monophyly of Blephariceromorpha. Instead, all analyses strongly supported Deuterophlebiidae and Nymphomyiidae as forming the basal-most position of Diptera, while placing Blephariceridae within Psychodomorpha. The amino-acid-based phylogenies (Figure 3 and Figure 4) supported a well-resolved topology for Diptera, showing the following: (1) Culicomorpha diverged, representing the most basal lineage except Deuterophlebiidae + Nymphomyiidae; (2) a sister relationship between Tipulomorpha and Ptychopteromorpha; and (3) these successive clades collectively forming the sister group to the remaining Diptera. In contrast, nucleotide-based analyses produced a less robust alternative topology (bootstrap support <90% for key nodes), with (Tipulomorpha + Ptychopteromorpha) diverged first, followed by a clade comprising Culicomorpha sister to remaining dipteran groups.

**Figure 5:**
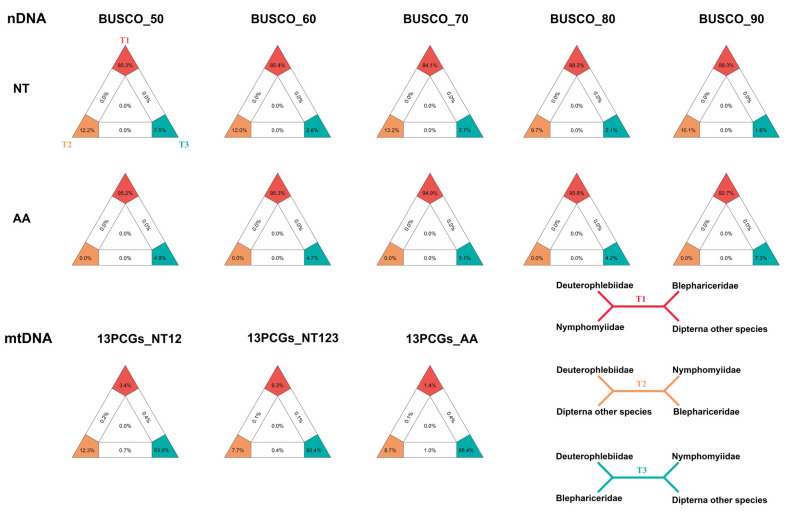
Four-cluster likelihood mapping (FcLM) analysis of concatenated nucleotide and amino acid alignments (nuclear/mitochondrial gene datasets). Topologies T1–T3 represent potential relationships among Blephariceridae, Deuterophlebiidae, and Nymphomyiidae. Percentages at vertices indicate fully resolved quartets across all possible groupings.

We employed DiscoVista to explore potential phylogenetic discordance between amino acid and nucleotide datasets. The results strongly supported most major dipteran clades but rejected the monophyly of Blephariceromorpha (Figure 4). Specifically, when Blephariceridae was grouped within Psychodomorpha, and Deuterophlebiidae, and Nymphomyiidae were positioned as the basal-most lineage, the phylogenetic trees received robust support. The comparative analysis revealed amino acid trees consistently recovered established dipteran suborders and most infraorders. In contrast, nucleotide trees failed to support monophyly for several groups including Culicomorpha, Bibionomorpha, and Psychodomorpha. This demonstrates that amino-acid-based analyses were more reliable in terms of node support and topological consistency. In addition, we further employed FcLM to examine the relationships among Blephariceridae, Deuterophlebiidae, and Nymphomyiidae within the broader context of dipteran groups (Figure 5). The nuclear gene datasets indicated a closer relationship between Deuterophlebiidae and Nymphomyiidae, while Blephariceridae appeared more closely related to other dipteran groups, particularly in the amino acid datasets.

Finally, we generated consensus trees from our amino acid and nucleotide datasets using DensiTree (v 3.1.0) (Figures S2 and S3). While the amino-acid-based and nucleotide-based trees exhibited distinct topologies, they both consistently identified the basal-most position of Diptera as a clade consisting of (Deuterophlebiidae + Nymphomyiidae). This consistency highlights the reliability of nuclear genomic data in resolving early branching events in Diptera evolution.

### 2.3. Phylogenetic Analyses Using Mitochondrial Datasets

Phylogenetic analyses were performed using mitochondrial genomes from 114 species, including our newly sequenced 11 Blephariceromorpha species. While the monophyly of each family (Blephariceridae, Deuterophlebiidae, and Nymphomyiidae) was strongly supported, relationships among higher taxa showed inconsistencies and low support (bootstrap support <80% for key nodes; Figure S4, Supplementary Files). Notably, the phylogenetic positions of Blephariceridae, Deuterophlebiidae, and Nymphomyiidae exhibited significant variations across the datasets.

To further assess the consistency of the phylogenetic trees, we used DiscoVista to test specific hypotheses concerning the monophyly of Blephariceromorpha and the potential placement of Deuterophlebiidae and Nymphomyiidae as basal lineages of Diptera (Figure S4). The analyses rejected monophyly for most traditionally defined suborders and infraorders of Diptera, including Blephariceromorpha, aligning with the weak nodal bootstrap support observed. Additionally, likelihood mapping analysis (Figure 5), which examined relationships between Blephariceridae, Deuterophlebiidae, Nymphomyiidae, and other dipteran taxa, revealed a topology where Blephariceridae and Deuterophlebiidae formed a group, while Nymphomyiidae appeared more closely related to the remaining Diptera.

Attempts to visualize the mitochondrial trees using DensiTree (v 3.1.0) to derive a consensus tree were unsuccessful. Consequently, the relationships among the three families within Blephariceromorpha and the basal lineages of Diptera remain unresolved, highlighting the persistent challenges requiring complementary nuclear genomic approaches to clarify these basal dipteran relationships.

## 3. Discussion

### 3.1. New Insights into Phylogeny of Nematocera

This study reconstructed dipteran phylogeny, with a particular focus on Nematocera, using a comprehensive dataset representing 64 families (>100 species) with Mecoptera and Siphonaptera as outgroups. Our transcriptome assembly quality was rigorously assessed using the diptera_odb10 BUSCO set (v5.6.1) [28], which currently provides the most comprehensive benchmark for dipteran phylogenomics (3285 genes from 56 species). For our focal taxa, RNA-Seq data yielded over 1500 high-confidence single-copy orthologs per species at 80% occupancy thresholds, collectively providing phylogenomic-grade data comparable to genome-derived markers.

The results demonstrated that nuclear genomic data yield superior phylogenetic inferences, with amino-acid-based trees consistently recovering monophyletic major clades (Figure 3). To explore these inconsistencies, we employed FcLM and DiscoVista (v1.0) to assess discordances across datasets and tree topologies. While mitochondrial genomes remain valuable for phylogenetic studies at shallow taxonomic levels due to their compact size, maternal inheritance, and elevated mutation rates [23,29,30], our analyses revealed significant limitations at deeper evolutionary timescales. Mitochondrial gene trees exhibited artifacts including long-branch attraction and substitution saturation [31,32,33,34], whereas nuclear genomic datasets demonstrated a superior phylogenetic performance, owing to their multi-locus nature and lower susceptibility to systematic biases. These findings corroborated an emerging consensus that genome-scale nuclear data provide more reliable phylogenetic signal for deep divergences [35,36,37]. Comparative analyses further showed that amino acid sequences outperformed nucleotide data by producing higher clade support values (FcLM/DiscoVista) and showing greater topological consistency. This aligns with the established understanding that amino acid substitutions are less affected by saturation and compositional heterogeneity, both of which are known to compromise phylogenetic accuracy [26,38,39,40].

By integrating these findings, we constructed a highly supported phylogenetic tree for Diptera, with a particular focus on the suborder Nematocera. The monophyly of each dipteran family was consistently reinforced, and relationships within the suborder showed variation depending on the dataset and method used. Earlier studies of Nematocera phylogeny, excluding Deuterophlebiidae and Nymphomyiidae, proposed a topology based on morphological evidence: (Culicomorpha + (Tipulomorpha + ((Blephariceridae + Tanyderidae) + ((Psychodidae + (Trichoceridae + Bibionomorpha)) + Brachycera)))) [2]. Another study, which combined morphological and molecular data, suggested the following widely accepted topology: (Tipulomorpha + (Psychodomorpha + (Culicomorpha + (Bibionomorpha + Brachycera)))) [18]. In addition, a comprehensive Insecta Phylogenomic study revealed an alternative topology in which Culicomorpha diverged earlier than Tipulomorpha [24].

In our study, which used similar methodologies to the Insecta Phylogenomics by employing large nuclear gene datasets for phylogenetic analysis of Diptera, we found that amino acid data supported the topology ((Deuterophlebiidae + Nymphomyiidae) + (Culicomorpha + ((Ptychopteromorpha + Tipulomorpha) + (Psychodomorpha + (Bibionomorpha + Brachycera))))), while nucleotide data produced results more consistent with classical dipteran phylogenies (Figure 3, Figure 4, Figures S3 and S4 Supplementary Files).

While chromosome-level genomes remain ideal, their current unavailability for Deuterophlebiidae, Nymphomyiidae, and Blephariceridae highlights the importance of our transcriptome-based approach. Despite significant progress in Diptera genomics, basal nematoceran lineages continue to represent a critical sampling gap in current genomic resources. To address this limitation, we recommend that future studies incorporate the newly released BUSCO sets (diptera_odb12 and nematocera_odb12) [41] with RNA-Seq data, as our results demonstrate that transcriptome sequencing provides a phylogenomic-grade resolution for non-model organisms [25]. Our comprehensive analyses demonstrate that nuclear gene-based phylogenetic trees exhibit remarkable consistency across analytical methods when using amino acid sequences, in contrast to the methodological sensitivity observed in nucleotide-based approaches [26,38,39,40]. As a result, we present a novel topology for Diptera, offering broader taxonomic and gene representation compared to previous studies.

### 3.2. Investigation of Basal Lineages in Diptera

Our research placed particular emphasis on elucidating the phylogenetic positions of basal dipteran lineages, with special attention to the infraorder Blephariceromorpha. Traditional taxonomic classifications have proposed that Blephariceromorpha comprises Deuterophlebiidae, Nymphomyiidae, and Blephariceridae, based on several lines of evidence including shared ecological characteristics, overlapping geological distributions, and morphological features [13,15,42]. Nevertheless, these studies, often relying on morphological data or limited nuclear and mitochondrial gene datasets, have yielded inconsistent and debated conclusions regarding the positions of basal lineages and the monophyly of Blephariceromorpha.

Emerging phylogenetic studies have begun to clarify the positions of these enigmatic basal dipteran lineages, suggesting that Deuterophlebiidae represents a basal lineage of Diptera, while Blephariceridae is placed within Psychodomorpha [2,14]. However, the phylogenetic position of Nymphomyiidae remains unresolved. Previous morphological phylogenetic studies, limited by the lack of Deuterophlebiidae samples, supported Nymphomyiidae as a basal lineage of Diptera [18]. In addition, Wiegmann et al. proposed a sister-group relationship between (Deuterophlebiidae + (Nymphomyiidae + other Diptera species)) [2], while recent studies using mitochondrial genomes suggest that Nymphomyiidae belongs within Culicomorpha [14,20]. These conflicting results highlight the challenges in resolving the phylogenetic relationships of the basal dipteran lineages.

In our study, largely consistent with previous findings, the monophyly of Blephariceromorpha was rejected. We found that Blephariceridae is more closely aligned with Psychodomorpha, while Nymphomyiidae and Deuterophlebiidae form a sister clade, positioned as the basal lineage of Diptera. This conclusion is supported by morphological evidence, such as the loss of mouthparts, elongation of the terminal antennal segment, and the presence of sucker-baring larval prolegs adapted to fast-flowing streams in both Deuterophlebiidae and Nymphomyiidae, indicating a close evolutionary relationship between these two families [19].

Our study reconciles earlier morphological and molecular phylogenetic perspectives, offering a unified understanding of the evolutionary relationships within Diptera. By integrating comprehensive molecular datasets, including complete mitochondrial genomes and transcriptomic data, our research represents a significant advancement in dipteran phylogeny. This robust approach provides a more detailed and reliable phylogenetic analysis, overcoming the limitations of previous studies that relied on smaller, less informative datasets.

### 3.3. Convergent Evolution of Larval Morphology in Blephariceromorpha

The three families within the traditionally defined infraorder Blephariceromorpha—Blephariceridae, Deuterophlebiidae, and Nymphomyiidae—exhibit striking similarities in their larval morphological adaptations (e.g., prolegs) to fast-flowing stream environments. These shared ecological specializations, compounded with overlapping geological distribution, have historically justified their grouping. A central question in Dipteran phylogeny has been whether these similarities result from a single evolutionary origin or multiple instances of convergent evolution.

Our comprehensive molecular analysis provides decisive evidence on this question. Phylogenetic trees derived from nuclear datasets support the paraphyly of Blephariceromorpha, indicating that Deuterophlebiidae and Nymphomyiidae share a common ancestor with similar larval adaptations, while Blephariceridae likely evolved these traits independently. This pattern strongly suggests that the demanding elective pressures of fast-flowing stream environments have driven convergent evolution. Key adaptations like specialized prolegs for substrate attachment and streamlined body shapes appear to have emerged multiple times under similar ecological constraints. This supports broader theories of convergent evolution, where ecological niches often drive the repeated evolution of similar traits, even in distantly related species [43,44,45]. Notable examples include fang evolution in frogs [46], the springboard trapping mechanism in carnivorous pitcher plants [47], the transition from egg-laying to live-bearing in marine snails [48], and the compound eyes in the chitons [49]. In the case of Blephariceromorpha, the similar larval morphologies exemplify morphological and ecological convergence, specifically highlighting adaptations such as abdominal suckers and spinule-equipped prolegs. However, Blephariceridae larvae possess abdominal suckers combined with non-suckered prolegs, while those of Deuterophlebiidae and Nymphomyiidae exhibit spinule-equipped prolegs. This suggests that the origin of these structures, adapted for attachment in fast-flowing environments, reflects non-homologous but functionally analogous traits, highlighting their similar ecological roles despite different developmental origins.

A deeper understanding of the developmental mechanisms and gene regulatory networks underlying the convergent evolution of larval adaptations in fast-flowing environments is crucial to fully elucidating the evolutionary processes shaping these lineages. Investigating developmental pathways, such as gene regulation and morphogenetic patterns, could further reveal how different lineages independently evolved similar traits in response to parallel environmental pressures.

## 4. Materials and Methods

### 4.1. Sample Collection

Specimens of Diptera were collected from Yaowang Mountain, Zhejiang, China, and Wuyi Mountains, Fujian, China (Table 1). Fresh samples were preserved in RNAlater^®^ (Thermo Fisher Scientific, Waltham, MA, USA) for RNA extraction and in 70% ethanol for DNA extraction. The samples were stored at −80 °C (School of Life Sciences, Westlake University, Hangzhou, Zhejiang Province, China).

### 4.2. DNA Extraction, Mitogenomes Sequencing, and Analysis

The genomic DNA was extracted from undissected whole-body specimens of each species using the Qiagen DNeasy Blood and Tissue kit (Hilden, Germany), following the manufacturer’s protocol. Illumina Truseq libraries (San Diego, CA, USA) were prepared for each species, with an average insert size of 400 base pairs (bp). These libraries were then sequenced on the Illumina NovaSeq 6000 platform, generating 150 bp paired-end reads.

Adaptor sequences and low-quality reads were trimmed with Trimmomatic (v0.39) [50]. The mitochondrial genome of each species was assembled and annotated using Getorganelle (v1.7.7) [51], MITOS2 (v2.1.0) [52] and Mitofinder (v1.4.1) [53].

### 4.3. RNA Extraction, Sequencing, and Transcriptome Assembly

Total RNA was extracted from RNAlater-preserved samples using TRIzol (Thermo Fisher Scientific, Waltham, MA, USA). RNA sequencing libraries were prepared using the NEBNext Ultra RNA Library Prep Kit for Illumina (New England Biolabs, Ipswich, MA, USA), following the manufacturer’s instructions. RNA-seq libraries were sequenced on the Illumina HiSeq 2000 platform, generating 150 bp paired-end reads.

Raw sequencing reads were quality-trimmed using Trimmomatic (v0.39) [50], and transcriptome assembly was performed with Trinity (v2.15.1) [54]. The longest isoform per locus was extracted. The transcriptome assembly quality was assessed with BUSCO v5.6.1 (--mode transcriptome) [28], using the Diptera-specific ortholog set (diptera_odb10). This benchmark dataset comprises 3285 single-copy BUSCO genes conserved in 56 dipteran species (Eukaryota domain), with maximum intron length (≤130 kb) and sequence length (≤160 kb) thresholds.

### 4.4. Matrix Construction

Phylogenetic trees were reconstructed based on two datasets: mitochondrial genomes (114 species) [14,55,56,57,58,59,60,61,62,63,64,65,66,67,68,69,70,71,72,73,74,75,76,77,78,79,80,81,82,83,84,85,86,87,88,89,90,91,92,93,94,95,96,97,98,99,100,101,102] and nuclear genes (126 species) [103,104,105,106,107,108,109,110,111,112,113,114,115,116,117,118,119,120,121,122,123,124,125,126,127,128,129,130,131,132,133,134,135], both including 61 families of Diptera (Tables S3 and S4). The outgroup taxa (Mecoptera and Siphonaptera) were selected based on their well-established sister-group relationship with Diptera in molecular phylogenies [2,24], providing appropriate phylogenetic distance while minimizing long-branch artifacts.

For mitogenomes, the data analysis was conducted in PhyloSuite (v1.2.3) [136]. Sequences of protein-coding genes (PCGs) and RNA genes were aligned individually via the MAFFT (v7.520) [137]. The alignments were manually reviewed in MEGA (7.0) [138], and poorly aligned regions were removed using GBLOCKS (v.0.91b) [139]. The individual gene alignments were then concatenated with SEQUENCEMATRIX (v.1.8) [140] to generate the following three datasets: (1) 13PCGs_NT12 matrix, including the first and second codon positions of 13 PCGs (7360 sites); (2) 13PCGs_NT123 matrix, including all three codon positions of 13 PCGs (total of 11,040 sites); and (3) 13PCGs_AA matrix, including the amino acids of 13 PCGs (3555 sites).

For the nuclear genes, universal single-copy ortholog (USCO) sequences were extracted following the workflow “script2_BUSCO_extraction.sh” in PLWS (v1.0.6) [141]. Briefly, coding regions were first identified from nucleotide sequences and translated into protein sequences using TransDecoder (v5.5.0) [142]. Protein sequences were then aligned with MAFFT (v7.520) [137], and low-quality or highly variable regions were trimmed using TrimAl (v1.4.1) [143]. Finally, the refined protein alignments were concatenated into a single dataset using FASconCAT-G (v1.05.1) [144]. Five phylogenetic matrices were generated with strict per-locus species coverage thresholds (50%, 60%, 70%, 80%, and 90%), where each retained locus contained sequences from ≥the specified percentage of species. Separate nucleotide and amino acid datasets were constructed for each threshold (Table 2).

### 4.5. Phylogenetic Analysis

Phylogenetic analyses of the mitogenome datasets were conducted using Bayesian inference (BI), maximum likelihood (ML), and ASTRAL-III methods. For the nuclear gene datasets, we employed ML and ASTRAL-III for tree construction.

Maximum Likelihood (ML) analyses were performed using IQ-TREE (v2.3.4) [145]. ModelFinder (-m MFP --mset GTR, LG) [146] was utilized to select the optimal partitioning scheme and substitution models for both nucleotide and amino acid data (GTR for nucleotides, LG for amino acids). Alternatively, the GHOST model (-m GTR+FO+H4, LG+FO+H4) [147] was applied to address heterotachy (site-specific evolutionary rate variation across branches). Phylogenetic reconstructions were performed with ultrafast bootstrap approximation (UFBoot) [148] using 1000 replicates (-B 1000). Branch support was further strengthened by incorporating the SH-aLRT test (-alrt 1000) [149] and refining the trees through branch nearest-neighbor interchange (--bnni). Additionally, the rcluster algorithm (-rcluster 10) [150] with a threshold of 10 was employed to enhance both tree accuracy and efficiency.

Individual gene trees were inferred with IQ-TREE with automatically selected best models and analyzed with ASTRAL-III (v5.6.1) [151] to infer the coalescent-based species trees, with local branch supports estimated from quartet frequencies.

Bayesian inference (BI) was conducted using PhyloBayes MPI (v.1.9) [152] under the site-heterogeneous mixture models CAT + GTR or CAT + LG, with a discrete gamma distribution and four rate categories for nucleotide or amino acid datasets. Two independent Markov Chain Monte Carlo (MCMC) chains were run until convergence was achieved (maxdiff < 0.1). After discarding the first 25% of trees as burn-in, a consensus tree was computed from the remaining trees combined from both runs.

Tree visualization and modifications were performed using the ITOL web server (v6) [153], Figtree (v1.4.4) [154], ggtree (v3.10.0) [155], and Adobe Illustrator (v2022).

### 4.6. Analyses of Phylogenetic Discordance and Alternative Relationships

DiscoVista (v1.0) [156] was used to quantify and visualize discordance among phylogenetic trees for alternative topologies. Our analysis primarily focused on the monophyly of Diptera groups, with particular emphasis on Blephariceromorpha, using a bootstrap threshold of 95 (-t 95).

To further investigate potential incongruence or confounding signals within the nuclear and mitochondrial gene datasets, especially regarding the relationships among Blephariceridae, Deuterophlebiidae, and Nymphomyiidae, we applied four-cluster likelihood mapping (FcLM) [157] implemented in IQ-TREE (-lmap, -m GTR for nucleotides, LG for amino acids) [145] to display support for the three possible quartet topologies and visualize the strength of each alternative relationship.

## 5. Conclusions

Overall, this study provides a comprehensive analysis of the phylogenetic relationships within Diptera, particularly the basal lineages, utilizing a combination of mitochondrial and nuclear gene datasets. While some topological inconsistencies emerged between different analytical approaches and data types, several key findings received robust support. The majority of our phylogenetic reconstructions decisively rejected the monophyly of Blephariceromorpha. Notably, the findings establish Deuterophlebiidae + Nymphomyiidae as the basal-most lineage of and the sister group to the rest of Diptera, whereas Blephariceridae was found to be grouped within the Psychodomorpha, a clade distantly related to Deuterophlebiidae and Nymphomyiidae. This novel phylogenetic arrangement suggests convergent evolution as the underlying mechanism for the remarkably similar larval adaptations observed in these two groups, Blephariceridae and (Deuterophlebiidae + Nymphomyiidae). These insights contribute to our understanding of the evolutionary history and diversification of Diptera, highlighting the importance of comprehensive molecular datasets and rigorous phylogenetic analyses in resolving complex evolutionary relationships.

## Figures and Tables

**Table 1:** Species sampling information for this study.

Family	Species	Sampling Localities	mtDNA Accession	Transcriptomes Accession	Sample Sites
Blephariceridae	*Blepharicera* sp1.-QZ	Yaowang Mountain, Zhejiang, China	PQ463199	SRR32672063	28°45′25.73″ N, 118°58′34.78″ E
Blephariceridae	*Blepharicera* sp2.-WYM	Wuyi Mountains, Fujian, China	PQ463200	SRR32672062	27°36′15.86″ N, 117°47′03.92″ E
Blephariceridae	Blephariceridae sp1.-WYM	Wuyi Mountains, Fujian, China	PQ463201	SRR32672061	27°36′05.30″ N, 117°43′56.06″ E
Blephariceridae	*Philorus* sp1.-QZ	Yaowang Mountain, Zhejiang, China	PQ463208	SRR32672057	28°45′25.73″ N, 118°58′34.78″ E
Blephariceridae	*Philorus* sp2.-WYM	Wuyi Mountains, Fujian, China	PQ463209	SRR32672056	27°36′05.30″ N, 117°43′56.06″ E
Deuterophlebiidae	*Deuterophlebia acutirhina*	Wuyi Mountains, Fujian, China	PQ463202	SRR32672060	27°36′05.30″ N, 117°43′56.06″ E
Deuterophlebiidae	*Deuterophlebia* sp.-Dxp	Linzhi, Xizang, China	PQ463203	N/A	29°35′46.15″ N, 94°21′19.52″ E
Deuterophlebiidae	*Deuterophlebia* sp.-DY	Ailao Mountain, Yunnan, China	PQ463204	N/A	23°58′13.20″ N, 101°31′37.73″ E
Deuterophlebiidae	*Deuterophlebia* sp.-QZ	Yaowang Mountain, Zhejiang, China	PQ463205	N/A	28°45′25.73″ N, 118°58′34.78″ E
Deuterophlebiidae	*Deuterophlebia wuyiensis*	Wuyi Mountains, Fujian, China	PQ463206	SRR32672059	27°44′55.52″ N, 117°40′40.77″ E
Nymphomyiidae	*Nymphomyia* sp.-WYM	Wuyi Mountains, Fujian, China	PQ463207	SRR32672058	27°36′15.86″ N, 117°47′03.92″ E

**Table 2:** Loci and site information for amino acids and nucleotides in mitochondrial and nuclear gene datasets used for phylogenetic analysis.

Type 1	Type 2	Loci	Site	Description	Abbreviations
mtDNA	NT	13	7360	13PCGs, codon 1 and 2	13PCG_NT12
mtDNA	NT	13	11,040	13PCGs, codon 1, 2 and 3	13PCG_NT123
mtDNA	AA	13	3555	13PCGs	13PCG_AA
nDNA	AA	2931	1,344,362	Taxon occupancy 50%	AA_Matrix50
nDNA	AA	2648	1,167,399	Taxon occupancy 60%	AA_Matrix60
nDNA	AA	2204	890,185	Taxon occupancy 70%	AA_Matrix70
nDNA	AA	1533	546,636	Taxon occupancy 80%	AA_Matrix80
nDNA	AA	469	133,932	Taxon occupancy 90%	AA_Matrix90
nDNA	NT	2931	4,033,086	Taxon occupancy 50%	NT_Matrix50
nDNA	NT	2648	3,502,197	Taxon occupancy 60%	NT_Matrix60
nDNA	NT	2204	2,670,555	Taxon occupancy 70%	NT_Matrix70
nDNA	NT	1533	1,639,908	Taxon occupancy 80%	NT_Matrix80
nDNA	NT	469	401,796	Taxon occupancy 90%	NT_Matrix90

## Data Availability

Supplementary mtDNA analysis, supplementary mtDNA trees and supplementary nuclear trees (https://figshare.com/s/aa43c69df909a20b0f66, accessed on 6 May 2025).

## Supplementary Materials

The following supporting information can be downloaded at: https://www.mdpi.com/article/10.3390/ijms26125714/s1.

## References

[1] Systema Dipterorum. http://www.diptera.org/.

[2] Wiegmann B.M., Trautwein M.D., Winkler I.S., Barr N.B., Kim J.-W., Lambkin C., Bertone M.A., Cassel B.K., Bayless K.M., Heimberg A.M. (2011). Episodic Radiations in the Fly Tree of Life. Proc. Natl. Acad. Sci. USA.

[3] Yeates D., Wiegmann B. (2005). Phylogeny and Evolution of Diptera: Recent Insights and New Perspectivs. The Evolutionary Biology of Flies.

[4] Yeates D.K., Wiegmann B.M., Courtney G.W., Meier R., Lambkin C., Pape T. (2007). Phylogeny and Systematics of Diptera: Two Decades of Progress and Prospects. Zootaxa.

[5] Yeates D., Wiegmann B. (2017). Phylogeny of Diptera. Manual of Afrotropical Diptera.

[6] Borkent A. (2018). The State of Phylogenetic Analysis: Narrow Visions and Simple Answers-Examples from the Diptera (Flies). Zootaxa.

[7] Savage J., Borkent A., Brodo F., Cumming J.M., Curler G., Currie D.C., de Waard J.R., Gibson J.F., Hauser M., Laplante L. (2019). Diptera of Canada. ZooKeys.

[8] Gerhardt R.R., Hribar L.J., Mullen G.R., Durden L.A. (2019). Chapter 11—Flies (Diptera). In Medical and Veterinary Entomology.

[9] Hayon I., Mendel Z., Dorchin N. (2016). Predatory Gall Midges on Mealybug Pests—Diversity, Life History, and Feeding Behavior in Diverse Agricultural Settings. Biol. Control.

[10] Weaver S.C., Reisen W.K. (2010). Present and Future Arboviral Threats. Antivir. Res..

[11] Wallace J.B., Webster J.R. (1996). The Role of Macroinvertebrates in Stream Ecosystem Function. Annu. Rev. Entomol..

[12] Orford K.A., Vaughan I.P., Memmott J. (2015). The Forgotten Flies: The Importance of Non-Syrphid Diptera as Pollinators. Proc. R. Soc. B Biol. Sci..

[13] Oosterbroek P., Courtney G. (1995). Phylogeny of the Nematocerous Families of Diptera (Insecta). Zool. J. Linn. Soc..

[14] Zhang X., Yang D., Kang Z. (2023). New Data on the Mitochondrial Genome of Nematocera (Lower Diptera): Features, Structures and Phylogenetic Implications. Zool. J. Linn. Soc..

[15] Gregory W. (1991). Courtney Phylogenetic Analysis of the Blephariceromorpha, with Special Reference to Mountain Midges (Diptera: Deuterophlebiidae. Syst. Entomol..

[16] Wood D.M. (1989). Phylogeny and Classification of the Nenatocera. Man. Nearctic Diptera.

[17] Bertone M.A., Courtney G.W., Wiegmann B.M. (2008). Phylogenetics and Temporal Diversification of the Earliest True Flies (Insecta: Diptera) Based on Multiple Nuclear Genes. Syst. Entomol..

[18] Lambkin C.L., Sinclair B.J., Pape T., Courtney G.W., Skevington J.H., Meier R., Yeates D.K., Blagoderov V., Wiegmann B.M. (2013). The Phylogenetic Relationships among Infraorders and Superfamilies of Diptera Based on Morphological Evidence. Syst. Entomol..

[19] Schneeberg K., Courtney G.W., Beutel R.G. (2011). Adult Head Structures of Deuterophlebiidae (Insecta), a Highly Derived “Ancestral” Dipteran Lineage. Arthropod Struct. Dev..

[20] Sæther O.A. (2000). Phylogeny of Culicomorpha (Diptera). Syst. Entomol..

[21] Kapli P., Yang Z., Telford M.J. (2020). Phylogenetic Tree Building in the Genomic Age. Nat. Rev. Genet..

[22] Yang Z., Bruce R. (2012). Molecular Phylogenetics: Principles and Practice. Nat. Rev. Genet..

[23] Cameron S.L. (2014). Insect Mitochondrial Genomics: Implications for Evolution and Phylogeny. Annu. Rev. Entomol..

[24] Misof B., Liu S., Meusemann K., Peters R.S., Donath A., Mayer C., Frandsen P.B., Ware J., Flouri T., Beutel R.G. (2014). Phylogenomics Resolves the Timing and Pattern of Insect Evolution. Science.

[25] Ge X., Peng L., Morse J.C., Wang J., Zang H., Yang L., Sun C., Wang B. (2024). Phylogenomics Resolves a 100-Year-Old Debate Regarding the Evolutionary History of Caddisflies (Insecta: Trichoptera). Mol. Phylogenet. Evol..

[26] Yu D., Ding Y., Tihelka E., Cai C., Hu F., Liu M., Zhang F. (2022). Phylogenomics of Elongate-Bodied Springtails Reveals Independent Transitions from Aboveground to Belowground Habitats in Deep Time. Syst. Biol..

[27] Du S., Tihelka E., Yu D., Chen W.-J., Bu Y., Cai C., Engel M.S., Luan Y.-X., Zhang F. (2024). Revisiting the Four Hexapoda Classes: Protura as the Sister Group to All Other Hexapods. Proc. Natl. Acad. Sci. USA.

[28] Manni M., Berkeley M.R., Seppey M., Simão F.A., Zdobnov E.M. (2021). BUSCO Update: Novel and Streamlined Workflows along with Broader and Deeper Phylogenetic Coverage for Scoring of Eukaryotic, Prokaryotic, and Viral Genomes. Mol. Biol. Evol..

[29] Boore J.L. (2006). The Use of Genome-Level Characters for Phylogenetic Reconstruction. Trends Ecol. Evol..

[30] Jeffrey L. (1999). Boore Animal Mitochondrial Genomes. Nucleic Acids Res..

[31] Song F., Li H., Jiang P., Zhou X., Liu J., Sun C., Vogler A.P., Cai W. (2016). Capturing the Phylogeny of Holometabola with Mitochondrial Genome Data and Bayesian Site-Heterogeneous Mixture Models. Genome Biol. Evol..

[32] Talavera G., Vila R. (2011). What Is the Phylogenetic Signal Limit from Mitogenomes? The Reconciliation between Mitochondrial and Nuclear Data in the Insecta Class Phylogeny. BMC Evol. Biol..

[33] Jeffroy O., Brinkmann H., Delsuc F., Philippe H. (2006). Phylogenomics: The Beginning of Incongruence?. Trends Genet..

[34] Bergsten J. (2005). A Review of Long-Branch Attraction. Cladistics.

[35] Feng S., Stiller J., Deng Y., Armstrong J., Fang Q., Reeve A.H., Xie D., Chen G., Guo C., Faircloth B.C. (2020). Dense Sampling of Bird Diversity Increases Power of Comparative Genomics. Nature.

[36] Stiller J., Feng S., Chowdhury A.-A., Rivas-González I., Duchêne D.A., Fang Q., Deng Y., Kozlov A., Stamatakis A., Claramunt S. (2024). Complexity of Avian Evolution Revealed by Family-Level Genomes. Nature.

[37] Uribe J.E., González V.L., Irisarri I., Kano Y., Herbert D.G., Strong E.E., Harasewych M.G. (2022). A Phylogenomic Backbone for Gastropod Molluscs. Syst. Biol..

[38] Cox C.J., Li B., Foster P.G., Embley T.M., Civáň P. (2014). Conflicting Phylogenies for Early Land Plants Are Caused by Composition Biases among Synonymous Substitutions. Syst. Biol..

[39] Shin S., Clarke D.J., Lemmon A.R., Moriarty Lemmon E., Aitken A.L., Haddad S., Farrell B.D., Marvaldi A.E., Oberprieler R.G., McKenna D.D. (2018). Phylogenomic Data Yield New and Robust Insights into the Phylogeny and Evolution of Weevils. Mol. Biol. Evol..

[40] Philippe H., Brinkmann H., Lavrov D.V., Littlewood D.T.J., Manuel M., Wörheide G., Baurain D. (2011). Resolving Difficult Phylogenetic Questions: Why More Sequences Are Not Enough. PLoS Biol..

[41] Tegenfeldt F., Kuznetsov D., Manni M., Berkeley M., Zdobnov E.M., Kriventseva E.V. (2025). OrthoDB and BUSCO Update: Annotation of Orthologs with Wider Sampling of Genomes. Nucleic Acids Res..

[42] Yeates D.K., Wiegmann B.M. (1999). CONGRUENCE AND CONTROVERSY: Toward a Higher-Level Phylogeny of Diptera. Annu. Rev. Entomol..

[43] Blount Z.D., Lenski R.E., Losos J.B. (2018). Contingency and Determinism in Evolution: Replaying Life’s Tape. Science.

[44] Balboa N., Shackelford T.K., Weekes-Shackelford V.A. (2021). Adaptation and Natural Selection. Encyclopedia of Evolutionary Psychological Science.

[45] Gould S.J. (1989). Wonderful Life: The Burgess Shale and the Nature of History.

[46] Sharon B., Emerson G. (2001). A Macroevolutionary Study of Historical Contingency in the Fanged Frogs of Southeast Asia. Biol. J. Linn. Soc..

[47] Chomicki G., Burin G., Busta L., Gozdzik J., Jetter R., Mortimer B., Bauer U. (2024). Convergence in Carnivorous Pitcher Plants Reveals a Mechanism for Composite Trait Evolution. Science.

[48] Stankowski S., Zagrodzka Z.B., Garlovsky M.D., Pal A., Shipilina D., Castillo D.G., Lifchitz H., Moan A.L., Leder E., Reeve J. (2024). The Genetic Basis of a Recent Transition to Live-Bearing in Marine Snails. Science.

[49] Varney R.M., Speiser D.I., Cannon J.T., Aguilar M.A., Eernisse D.J., Oakley T.H. (2024). A Morphological Basis for Path-Dependent Evolution of Visual Systems. Science.

[50] Bolger A.M., Lohse M., Usadel B. (2014). Trimmomatic: A Flexible Trimmer for Illumina Sequence Data. Bioinformatics.

[51] Jin J.-J., Yu W.-B., Yang J.-B., Song Y., de Pamphilis C.W., Yi T.-S., Li D.-Z. (2020). GetOrganelle: A Fast and Versatile Toolkit for Accurate de Novo Assembly of Organelle Genomes. Genome biol..

[52] Donath A., Jühling F., Al-Arab M., Bernhart S.H., Reinhardt F., Stadler P.F., Middendorf M., Bernt M. (2019). Improved Annotation of Protein-Coding Genes Boundaries in Metazoan Mitochondrial Genomes. Nucleic Acids Res..

[53] Allio R., Schomaker-Bastos A., Romiguier J., Prosdocimi F., Nabholz B., Delsuc F. (2020). MitoFinder: Efficient Automated Large-Scale Extraction of Mitogenomic Data in Target Enrichment Phylogenomics. Mol. Ecol. Resour..

[54] Grabherr M.G., Haas B.J., Yassour M., Levin J.Z., Thompson D.A., Amit I., Adiconis X., Fan L., Raychowdhury R., Zeng Q. (2011). Full-Length Transcriptome Assembly from RNA-Seq Data without a Reference Genome. Nat. Biotechnol..

[55] Lin X.-L., Liu Z., Yan L.-P., Duan X., Bu W.-J., Wang X.-H., Zheng C.-G. (2022). Mitogenomes Provide New Insights of Evolutionary History of Boreheptagyiini and Diamesini (Diptera: Chironomidae: Diamesinae). Ecol. Evol..

[56] Ren L., Guo Q., Yan W., Guo Y., Ding Y. (2016). The Complete Mitochondria Genome of *Calliphora vomitoria* (Diptera: Calliphoridae). Mitochondrial DNA Part B.

[57] Qi Y., Xu J., Tian X., Bai Y., Gu X. (2017). The Complete Mitochondrial Genome of *Hermetia illucens* (Diptera: Stratiomyidae). Mitochondrial DNA Part B.

[58] Liu Z.-Q., Kuermanali N., Li Z., Chen S.-J., Wang Y.-Z., Tao H., Chen C.-F. (2017). The Complete Mitochondrial Genome of the Parasitic Sheep Ked *Melophagus ovinus* (Diptera: Hippoboscidae). Mitochondrial DNA Part B.

[59] Tan L., Guan X., Zhang L., Zhu F., Lei C. (2018). The Complete Mitochondrial Genome of the Flea *Ceratophyllus wui* (Siphonaptera: Ceratophyllidae). Mitochondrial DNA Part B.

[60] Jiang X., Han X., Liu Q., Hou X. (2019). The Mitochondrial Genome of *Forcipomyia makanensis* (Insecta: Diptera: Ceratopogonidae). Mitochondrial DNA Part B.

[61] Tan L., Yao X., Liu J., Lei C., Huang Q., Hu B. (2023). The Complete Mitochondrial Genome of the Flea *Hystrichopsylla weida qinlingensis* (Siphonaptera: Hystrichopsylla). Mitochondrial DNA Part B.

[62] Cameron S.L., Lambkin C.L., Barker S.C., Whiting M.F. (2007). A Mitochondrial Genome Phylogeny of Diptera: Whole Genome Sequence Data Accurately Resolve Relationships over Broad Timescales with High Precision. Syst. Entomol..

[63] Lin X.-L., Zhao Y.-M., Yan L.-P., Liu W.-B., Bu W.-J., Wang X.-H., Zheng C.-G. (2022). Mitogenomes Provide New Insights into the Evolutionary History of Prodiamesinae (Diptera: Chironomidae). Zool. Scr..

[64] Milián-García Y., Hempel C.A., Janke L.A.A., Young R.G., Furukawa-Stoffer T., Ambagala A., Steinke D., Hanner R.H. (2022). Mitochondrial Genome Sequencing, Mapping, and Assembly Benchmarking for *Culicoides* Species (Diptera: Ceratopogonidae). BMC Genom..

[65] (2018). Li Xu-Dong; Chen Bin Sequencing and analysis of the complete mitochondrial genome of *Armigeres subalbatus* (Diptera: Culicidae). Acta Entomol. Sin..

[66] Li X., Li W., Ding S., Cameron S.L., Mao M., Shi L., Yang D. (2017). Mitochondrial Genomes Provide Insights into the Phylogeny of Lauxanioidea (Diptera: Cyclorrhapha). Int. J. Mol. Sci..

[67] Li H.-N., Pei W.-Y., Wang M.-F., Chen B.-Q., Peng H.-L., Cao R.-J., Zhao M.-T., Yang J., Zhang X.-C., Zhang D. (2023). Mitochondrial Genomes Provide New Insights into the Phylogeny and Evolution of Anthomyiidae (Insecta: Diptera). Arthropod Syst. Phylogeny.

[68] Li S.-Y., Chen M.-H., Sun L., Wang R.-H., Li C.-H., Gresens S., Li Z., Lin X.-L. (2024). New Mitogenomes from the Genus *Cricotopus* (Diptera: Chironomidae, Orthocladiinae): Characterization and Phylogenetic Implications. Arch. Insect Biochem. Physiol..

[69] Beliavskaia A., Tan K.-K., Sinha A., Husin N.A., Lim F.S., Loong S.K., Bell-Sakyi L., Carlow C.K.S., AbuBakar S., Darby A.C. (2023). Metagenomics of Culture Isolates and Insect Tissue Illuminate the Evolution of *Wolbachia*, *Rickettsia* and *Bartonella* Symbionts in *Ctenocephalides* spp. Fleas. Microb. Genom..

[70] Wong D., Norman H., Creedy T.J., Jordaens K., Moran K.M., Young A., Mengual X., Skevington J.H., Vogler A.P. (2023). The Phylogeny and Evolutionary Ecology of Hoverflies (Diptera: Syrphidae) Inferred from Mitochondrial Genomes. Mol. Phylogenet. Evol..

[71] Trinca V., Uliana J.V.C., Ribeiro G.K.S., Torres T.T., Monesi N. (2022). Characterization of the Mitochondrial Genomes of *Bradysia hygida*, *Phytosciara flavipes* and *Trichosia splendens* (Diptera: Sciaridae) and Novel Insights on the Control Region of Sciarid Mitogenomes. Insect Mol. Biol..

[72] Wang Q., Huang J., Wu H. (2021). Mitogenomes Provide Insights into the Phylogeny of Mycetophilidae (Diptera: Sciaroidea). Gene.

[73] Li X., Ding S., Li X., Hou P., Tang C., Yang D. (2017). The Complete Mitochondrial Genome Analysis of *Eristalis tenax* (Diptera, Syrphidae). Mitochondrial DNA Part B.

[74] Xiang H.-T., Wen F.-Q., Wang G.-L. (2017). The Complete Nucleotide Sequence of the Mitochondrial Genome of *Dorcadia ioffi* (Siphonaptera: Vermipsyllidae). Mitochondrial DNA Part B.

[75] Li N., Hu G.-L., Hua B.-Z. (2019). Complete Mitochondrial Genomes of *Bittacus strigosus* and *Panorpa debilis* and Genomic Comparisons of Mecoptera. Int. J. Biol. Macromol..

[76] Zhang X., Kang Z., Ding S., Wang Y., Borkent C., Saigusa T., Yang D. (2019). Mitochondrial Genomes Provide Insights into the Phylogeny of Culicomorpha (Insecta: Diptera). Int. J. Mol. Sci..

[77] Foster P.G., de Oliveira T.M.P., Bergo E.S., Conn J.E., Sant’Ana D.C., Nagaki S.S., Nihei S., Lamas C.E., González C., Moreira C.C. (2017). Phylogeny of Anophelinae Using Mitochondrial Protein Coding Genes. R. Soc. Open Sci..

[78] Battaglia V., Gabrieli P., Brandini S., Capodiferro M.R., Javier P.A., Chen X.-G., Achilli A., Semino O., Gomulski L.M., Malacrida A.R. (2016). The Worldwide Spread of the Tiger Mosquito as Revealed by Mitogenome Haplogroup Diversity. Front. Genet..

[79] Jiang F., Pan X., Li X., Yu Y., Zhang J., Jiang H., Dou L., Zhu S. (2016). The First Complete Mitochondrial Genome of *Dacus longicornis* (Diptera: Tephritidae) Using next-Generation Sequencing and Mitochondrial Genome Phylogeny of Dacini Tribe. Sci. Rep..

[80] Wang K., Li X., Ding S., Wang N., Mao M., Wang M., Yang D. (2016). The Complete Mitochondrial Genome of the *Atylotus miser* (Diptera: Tabanomorpha: Tabanidae), with Mitochondrial Genome Phylogeny of Lower Brachycera (Orthorrhapha). Gene.

[81] Zhang D., Yan L., Zhang M., Chu H., Cao J., Li K., Hu D., Pape T. (2016). Phylogenetic Inference of Calyptrates, with the First Mitogenomes for Gasterophilinae (Diptera: Oestridae) and Paramacronychiinae (Diptera: Sarcophagidae). Int. J. Biol. Sci..

[82] Gao D.-Z., Liu G.-H., Song H.-Q., Wang G.-L., Wang C.-R., Zhu X.-Q. (2016). The Complete Mitochondrial Genome of *Gasterophilus intestinalis*, the First Representative of the Family Gasterophilidae. Parasitol. Res..

[83] Deviatiiarov R., Kikawada T., Gusev O. (2017). The Complete Mitochondrial Genome of an Anhydrobiotic Midge *Polypedilum vanderplanki* (Chironomidae, Diptera). Mitochondrial DNA Part A.

[84] Briscoe A.G., Sivell D., Harbach R.E. (2017). The Complete Mitochondrial Genome of *Dixella aestivalis* (Diptera: Nematocera: Dixidae). Mitochondrial DNA Part A.

[85] Kim S., Kim H., Shin S.C. (2016). Complete Mitochondrial Genome of the Antarctic Midge *Parochlus Steinenii* (Diptera: Chironomidae). Mitochondrial DNA Part A.

[86] Zhang N.-X., Yu G., Li T.-J., He Q.-Y., Zhou Y., Si F.-L., Ren S., Chen B. (2015). The Complete Mitochondrial Genome of Delia Antiqua and Its Implications in Dipteran Phylogenetics. PLoS ONE.

[87] Ye F., Liu T., King S.D., You P. (2015). Mitochondrial Genomes of Two Phlebotomine Sand Flies, *Phlebotomus chinensis* and *Phlebotomus papatasi* (Diptera: Nematocera), the First Representatives from the Family Psychodidae. Parasites Vectors.

[88] Atray I., Bentur J.S., Nair S. (2015). The Asian Rice Gall Midge (*Orseolia oryzae*) Mitogenome Has Evolved Novel Gene Boundaries and Tandem Repeats That Distinguish Its Biotypes. PLoS ONE.

[89] Hardy C.M., Court L.N., Morgan M.J. (2016). The Complete Mitochondrial DNA Genome of *Aedes Vigilax* (Diptera: Culicidae). Mitochondrial DNA Part A.

[90] Li X., Wang Y., Su S., Yang D. (2016). The Complete Mitochondrial Genomes of *Musca domestica* and *Scathophaga stercoraria* (Diptera: Muscoidea: Muscidae and Scathophagidae). Mitochondrial DNA Part A.

[91] Zhong M., Wang X., Liu Q., Luo B., Wu C., Wen J. (2016). The Complete Mitochondrial Genome of the Scuttle Fly, *Megaselia scalaris* (Diptera: Phoridae). Mitochondrial DNA Part A.

[92] Zhong M., Wang X., Liu Q., Luo B., Wu C., Wen J. (2016). The Complete Mitochondrial Genome of the Flesh Fly, *Boettcherisca peregrine* (Diptera: Sarcophagidae). Mitochondrial DNA Part A.

[93] Li Q., Wei S., Shi M., Chen X.-X. (2015). The Complete Mitochondrial Genome of *Neopanorpa pulchra* (Mecoptera: Panorpidae). Mitochondrial DNA Part A.

[94] Cameron S.L. (2015). The Complete Mitochondrial Genome of a Flea, *Jellisonia amadoi* (Siphonaptera: Ceratophyllidae). Mitochondrial DNA Part A.

[95] Chen S., Oliveira M.T., Sanz A., Kemppainen E., Fukuoh A., Schlicht B., Kaguni L.S., Jacobs H.T. (2012). A Cytoplasmic Suppressor of a Nuclear Mutation Affecting Mitochondrial Functions in *Drosophila*. Genetics.

[96] Nelson L.A., Cameron S.L., Yeates D.K. (2011). The Complete Mitochondrial Genome of the Gall-Forming Fly, *Fergusonina taylori* Nelson and Yeates (Diptera: Fergusoninidae). Mitochondrial DNA Part A.

[97] Beckenbach A.T. (2012). Mitochondrial Genome Sequences of Nematocera (Lower Diptera): Evidence of Rearrangement Following a Complete Genome Duplication in a Winter Crane Fly. Genome Biol. Evol..

[98] Behura S.K., Lobo N.F., Haas B., de Bruyn B., Lovin D.D., Shumway M.F., Puiu D., Romero-Severson J., Nene V., Severson D.W. (2011). Complete Sequences of Mitochondria Genomes of *Aedes aegypti* and *Culex quinquefasciatus* and Comparative Analysis of Mitochondrial DNA Fragments Inserted in the Nuclear Genomes. Insect Biochem. Mol. Biol..

[99] Beckenbach A.T. (2011). Mitochondrial Genome Sequences of Representatives of Three Families of Scorpionflies (Order Mecoptera) and Evolution in a Major Duplication of Coding Sequence. Genome.

[100] Wang S., Lei Z., Wang H., Dong B., Ren B. (2011). The Complete Mitochondrial Genome of the Leafminer *Liriomyza trifolii* (Diptera: Agromyzidae). Mol. Biol. Rep..

[101] Beckenbach A.T., Joy J.B. (2009). Evolution of the Mitochondrial Genomes of Gall Midges (Diptera: Cecidomyiidae): Rearrangement and Severe Truncation of tRNA Genes. Genome Biol. Evol..

[102] Beard C.B., Hamm D.M., Collins F.H. (1993). The Mitochondrial Genome of the Mosquito Anopheles Gambiae: DNA Sequence, Genome Organization, and Comparisons with Mitochondrial Sequences of Other Insects. Insect Mol. Biol..

[103] Bingxin G. (2022). Chromosome-Level Genome Assembly of *Chironomus striatipennis* Kieffer Provides Insights into Benthic Adaptation and Metamorphosis Mechanism. TechRxiv.

[104] Guo H., Wang G., Zhang S.T., Huang M. (2018). Development of SSR Primers for *Simulium* (*Eusimulium*) *Angustipes* (Diptera: Simuliidae) Based on RNA-Seq Dataset. Acta Entomol. Sin..

[105] Driscoll T.P., Verhoeve V.I., Gillespie J.J., Johnston J.S., Guillotte M.L., Rennoll-Bankert K.E., Rahman M.S., Hagen D., Elsik C.G., Macaluso K.R. (2020). A Chromosome-Level Assembly of the Cat Flea Genome Uncovers Rampant Gene Duplication and Genome Size Plasticity. BMC Biol..

[106] Morales-Hojas R., Hinsley M., Armean I.M., Silk R., Harrup L.E., Gonzalez-Uriarte A., Veronesi E., Campbell L., Nayduch D., Saski C. (2018). The Genome of the Biting Midge *Culicoides sonorensis* and Gene Expression Analyses of Vector Competence for Bluetongue Virus. BMC Genom..

[107] Wang X., Xiong M., Lei C., Zhu F. (2015). The Developmental Transcriptome of the Synanthropic Fly *Chrysomya megacephala* and Insights into Olfactory Proteins. BMC Genom..

[108] Pauli T., Burt T.O., Meusemann K., Bayless K., Donath A., Podsiadlowski L., Mayer C., Kozlov A., Vasilikopoulos A., Liu S. (2018). New Data, Same Story: Phylogenomics Does Not Support Syrphoidea (Diptera: Syrphidae, Pipunculidae). Syst. Entomol..

[109] Narayanan Kutty S., Meusemann K., Bayless K.M., Marinho M.A.T., Pont A.C., Zhou X., Misof B., Wiegmann B.M., Yeates D., Cerretti P. (2019). Phylogenomic Analysis of Calyptratae: Resolving the Phylogenetic Relationships within a Major Radiation of Diptera. Cladistics.

[110] Ren L., Shang Y., Yang L., Wang S., Wang X., Chen S., Bao Z., An D., Meng F., Cai J. (2021). Chromosome-Level de Novo Genome Assembly of *Sarcophaga peregrina* Provides Insights into the Evolutionary Adaptation of Flesh Flies. Mol. Ecol. Resour..

[111] Husnik F., Hypsa V., Darby A. (2020). Insect-Symbiont Gene Expression in the Midgut Bacteriocytes of a Blood-Sucking Parasite. Genome Biol. Evol..

[112] Huang M., Kingan S., Shoue D., Nguyen O., Froenicke L., Galvin B., Lambert C., Khan R., Maheshwari C., Weisz D. (2024). Improved High Quality Sand Fly Assemblies Enabled by Ultra Low Input Long Read Sequencing. Sci. Data.

[113] Fu Y., Fang X., Xiao Y., Mao B., Xu Z., Shen M., Wang X. (2024). Two Chromosome-Level Genomes of *Smittia aterrima* and *Smittia pratorum* (Diptera, Chironomidae). Sci. Data.

[114] Liu P., Yang W., Kong L., Zhao S., Xie Z., Zhao Y., Wu Y., Guo Y., Xie Y., Liu T. (2023). A DBHS Family Member Regulates Male Determination in the Filariasis Vector Armigeres Subalbatus. Nat. Commun..

[115] Mahajan S., Bachtrog D. (2017). Convergent Evolution of Y Chromosome Gene Content in Flies. Nat. Commun..

[116] Lin M.-D., Chuang C.-H., Kao C.-H., Chen S.-H., Wang S.-C., Hsieh P.-H., Chen G.-Y., Mao C.-C., Li J.-Y., Jade Lu M.-Y. (2024). Decoding the Genome of Bloodsucking Midge *Forcipomyia taiwana* (Diptera: Ceratopogonidae): Insights into Odorant Receptor Expansion. Insect Biochem. Mol. Biol..

[117] Cohen C.M., Cole T.J., Brewer M.S. (2020). Pick Your Poison: Molecular Evolution of Venom Proteins in Asilidae (Insecta: Diptera). Toxins.

[118] Sontowski R., Poeschl Y., Okamura Y., Vogel H., Guyomar C., Cortesero A.-M., van Dam N.M. (2022). A High-Quality Functional Genome Assembly of *Delia radicum* L. (Diptera: Anthomyiidae) Annotated from Egg to Adult. Mol. Ecol. Resour..

[119] Urban J.M., Foulk M.S., Bliss J.E., Coleman C.M., Lu N., Mazloom R., Brown S.J., Spradling A.C., Gerbi S.A. (2021). High Contiguity de Novo Genome Assembly and DNA Modification Analyses for the Fungus Fly, *Sciara coprophila*, Using Single-Molecule Sequencing. BMC Genom..

[120] Zhao C., Escalante L.N., Chen H., Benatti T.R., Qu J., Chellapilla S., Waterhouse R.M., Wheeler D., Andersson M.N., Bao R. (2015). A Massive Expansion of Effector Genes Underlies Gall-Formation in the Wheat Pest *Mayetiola destructor*. Curr. Biol..

[121] Shen X., Jin J., Zhang G., Yan B., Yu X., Wu H., Yang M., Zhang F. (2024). The Chromosome-Level Genome Assembly of *Aphidoletes aphidimyza* Rondani (Diptera: Cecidomyiidae). Sci. Data.

[122] Zhang B., Han H.-B., Xu L.-B., Li Y.-R., Song M.-X., Liu A.-P. (2021). Transcriptomic Analysis of Diapause-Associated Genes in *Exorista civilis* Rondani (Diptera:Tachinidae). Arch. Insect Biochem. Physiol..

[123] Schmidt H., Hellmann S.L., Waldvogel A.-M., Feldmeyer B., Hankeln T., Pfenninger M. (2020). A High-Quality Genome Assembly from Short and Long Reads for the Non-Biting Midge *Chironomus riparius* (Diptera). G3.

[124] Mahar J.E., Shi M., Hall R.N., Strive T., Holmes E.C. (2020). Comparative Analysis of RNA Virome Composition in Rabbits and Associated Ectoparasites. J. Virol..

[125] Narayanan Kutty S., Wong W.H., Meusemann K., Meier R., Cranston P.S. (2018). A Phylogenomic Analysis of Culicomorpha (Diptera) Resolves the Relationships among the Eight Constituent Families. Syst. Entomol..

[126] Jia Z., Hasi S., Vogl C., Burger P.A. (2022). Genomic Insights into Evolution and Control of *Wohlfahrtia magnifica*, a Widely Distributed Myiasis-Causing Fly of Warm-Blooded Vertebrates. Mol. Ecol. Resour..

[127] Amaral D.T., Johnson C.H., Viviani V.R. (2021). RNA-Seq Analysis of the Blue Light-Emitting *Orfelia fultoni* (Diptera: Keroplatidae) Suggest Photoecological Adaptations at the Molecular Level. Comp. Biochem. Physiol. Part D: Genom. Proteom..

[128] Melotto G., Jones M.W., Bosley K., Flack N., Frank L.E., Jacobson E., Kipp E.J., Nelson S., Ramirez M., Walls C. (2023). The Genome of the Soybean Gall Midge (*Resseliella maxima*). G3.

[129] Anderson N., Jaron K.S., Hodson C.N., Couger M.B., Ševčík J., Weinstein B., Pirro S., Ross L., Roy S.W. (2022). Gene-Rich X Chromosomes Implicate Intragenomic Conflict in the Evolution of Bizarre Genetic Systems. Proc. Natl. Acad. Sci. USA.

[130] Yoshida Y., Shaikhutdinov N., Kozlova O., Itoh M., Tagami M., Murata M., Nishiyori-Sueki H., Kojima-Ishiyama M., Noma S., Cherkasov A. (2022). High Quality Genome Assembly of the Anhydrobiotic Midge Provides Insights on a Single Chromosome-Based Emergence of Extreme Desiccation Tolerance. NAR Genom. Bioinf..

[131] Generalovic T.N., McCarthy S.A., Warren I.A., Wood J.M.D., Torrance J., Sims Y., Quail M., Howe K., Pipan M., Durbin R. (2021). A High-Quality, Chromosome-Level Genome Assembly of the Black Soldier Fly (*Hermetia illucens* L.). G3.

[132] Sun X., Liu W., Li R., Zhao C., Pan L., Yan C. (2021). A Chromosome Level Genome Assembly of *Propsilocerus akamusi* to Understand Its Response to Heavy Metal Exposure. Mol. Ecol. Resour..

[133] Konganti K., Guerrero F.D., Schilkey F., Ngam P., Jacobi J.L., Umale P.E., Perez de Leon A.A., Threadgill D.W. (2018). A Whole Genome Assembly of the Horn Fly, *Haematobia irritans*, and Prediction of Genes with Roles in Metabolism and Sex Determination. G3.

[134] Vicoso B., Bachtrog D. (2015). Numerous Transitions of Sex Chromosomes in Diptera. PLoS Biol..

[135] Hoskins R.A., Carlson J.W., Wan K.H., Park S., Mendez I., Galle S.E., Booth B.W., Pfeiffer B.D., George R.A., Svirskas R. (2015). The Release 6 Reference Sequence of the *Drosophila melanogaster* Genome. Genome Res..

[136] Zhang D., Gao F., Jakovlić I., Zou H., Zhang J., Li W.X., Wang G.T. (2020). PhyloSuite: An Integrated and Scalable Desktop Platform for Streamlined Molecular Sequence Data Management and Evolutionary Phylogenetics Studies. Mol. Ecol. Resour..

[137] Katoh K., Standley D.M. (2013). MAFFT Multiple Sequence Alignment Software Version 7: Improvements in Performance and Usability. Mol. Biol. Evol..

[138] Kumar S., Stecher G., Tamura K. (2016). MEGA7: Molecular Evolutionary Genetics Analysis Version 7.0 for Bigger Datasets. Mol. Biol. Evol..

[139] Castresana J. (2000). Selection of Conserved Blocks from Multiple Alignments for Their Use in Phylogenetic Analysis. Mol. Biol. Evol..

[140] Vaidya G., Lohman D.J., Meier R. (2011). SequenceMatrix: Concatenation Software for the Fast Assembly of Multi-Gene Datasets with Character Set and Codon Information. Cladistics.

[141] Zhang F., Ding Y., Zhu C.D., Zhou X., Orr M.C., Scheu S., Luan Y.-X. (2019). Phylogenomics from Low-Coverage Whole-Genome Sequencing. Methods Ecol. Evol..

[142] TransDecoder. https://github.com/TransDecoder/TransDecoder.

[143] Capella-Gutiérrez S., Silla-Martínez J.M., Gabaldón T. (2009). trimAl: A Tool for Automated Alignment Trimming in Large-Scale Phylogenetic Analyses. Bioinformatics.

[144] Kück P., Longo G.C. (2014). FASconCAT-G: Extensive Functions for Multiple Sequence Alignment Preparations Concerning Phylogenetic Studies. Front. Zool..

[145] Nguyen L.-T., Schmidt H.A., Von Haeseler A., Minh B.Q. (2015). IQ-TREE: A Fast and Effective Stochastic Algorithm for Estimating Maximum-Likelihood Phylogenies. Mol. Biol. Evol..

[146] Kalyaanamoorthy S., Minh B.Q., Wong T.K.F., von Haeseler A., Jermiin L.S. (2017). ModelFinder: Fast Model Selection for Accurate Phylogenetic Estimates. Nat. Methods.

[147] Crotty S.M., Minh B.Q., Bean N.G., Holland B.R., Tuke J., Jermiin L.S., Haeseler A.V. (2019). GHOST: Recovering Historical Signal from Heterotachously Evolved Sequence Alignments. Syst. Biol..

[148] Minh B.Q., Nguyen M.A.T., Von Haeseler A. (2013). Ultrafast Approximation for Phylogenetic Bootstrap. Mol. Biol. Evol..

[149] Guindon S., Dufayard J.-F., Lefort V., Anisimova M., Hordijk W., Gascuel O. (2010). New Algorithms and Methods to Estimate Maximum-Likelihood Phylogenies: Assessing the Performance of PhyML 3.0. Syst. Biol..

[150] Lanfear R., Calcott B., Kainer D., Mayer C., Stamatakis A. (2014). Selecting Optimal Partitioning Schemes for Phylogenomic Datasets. BMC Evol. Biol..

[151] Zhang C., Rabiee M., Sayyari E., Mirarab S. (2018). ASTRAL-III: Polynomial Time Species Tree Reconstruction from Partially Resolved Gene Trees. BMC Bioinf..

[152] Lartillot N., Lepage T., Blanquart S. (2009). PhyloBayes 3: A Bayesian Software Package for Phylogenetic Reconstruction and Molecular Dating. Bioinformatics.

[153] Letunic I., Bork P. (2024). Interactive Tree of Life (iTOL) v6: Recent Updates to the Phylogenetic Tree Display and Annotation Tool. Nucleic Acids Res..

[154] Andrew Rambaut Figtree. https://tree.bio.ed.ac.uk/software/figtree/.

[155] Yu G., Smith D.K., Zhu H., Guan Y., Lam T.T.Y. (2017). Ggtree: An r Package for Visualization and Annotation of Phylogenetic Trees with Their Covariates and Other Associated Data. Methods Ecol. Evol..

[156] Sayyari E., Whitfield J.B., Mirarab S. (2018). DiscoVista: Interpretable Visualizations of Gene Tree Discordance. Mol. Phylogenet. Evol..

[157] Strimmer K., Von Haeseler A. (1997). Likelihood-Mapping: A Simple Method to Visualize Phylogenetic Content of a Sequence Alignment. Proc. Natl. Acad. Sci. USA.

