# Supplementary figures and images for: Genomic Insights into Basal Diptera Phylogeny: The Non-Monophyletic Nature of Blephariceromorpha
Source: Int J Mol Sci. 2025 Jun 14;26(12):5714. doi: 10.3390/ijms26125714 (PMC12192722; doi:10.3390/ijms26125714)

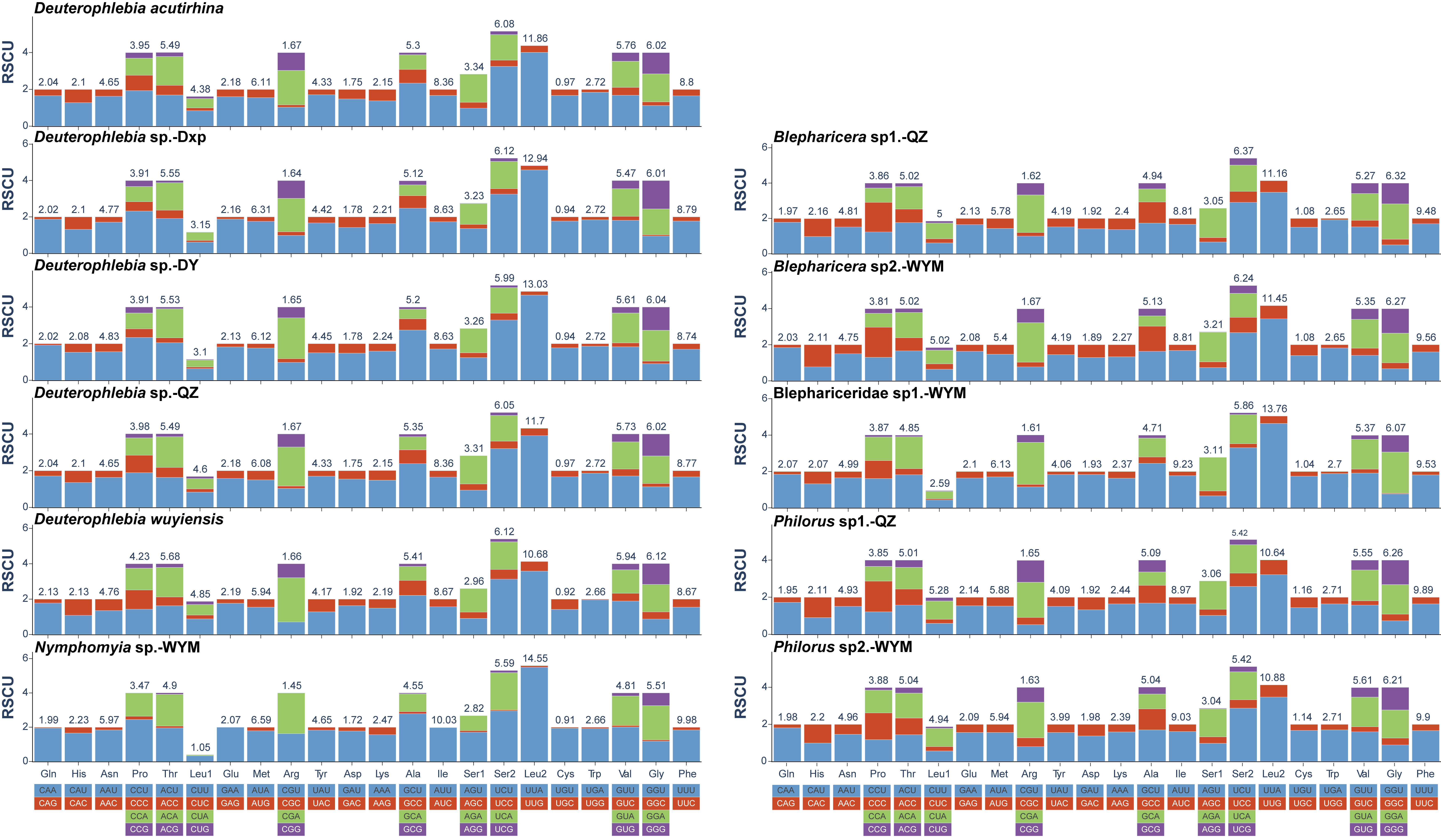

Supplement: Supplementary file 1 [file ijms-26-05714-s001.zip › Figure S1.jpg]

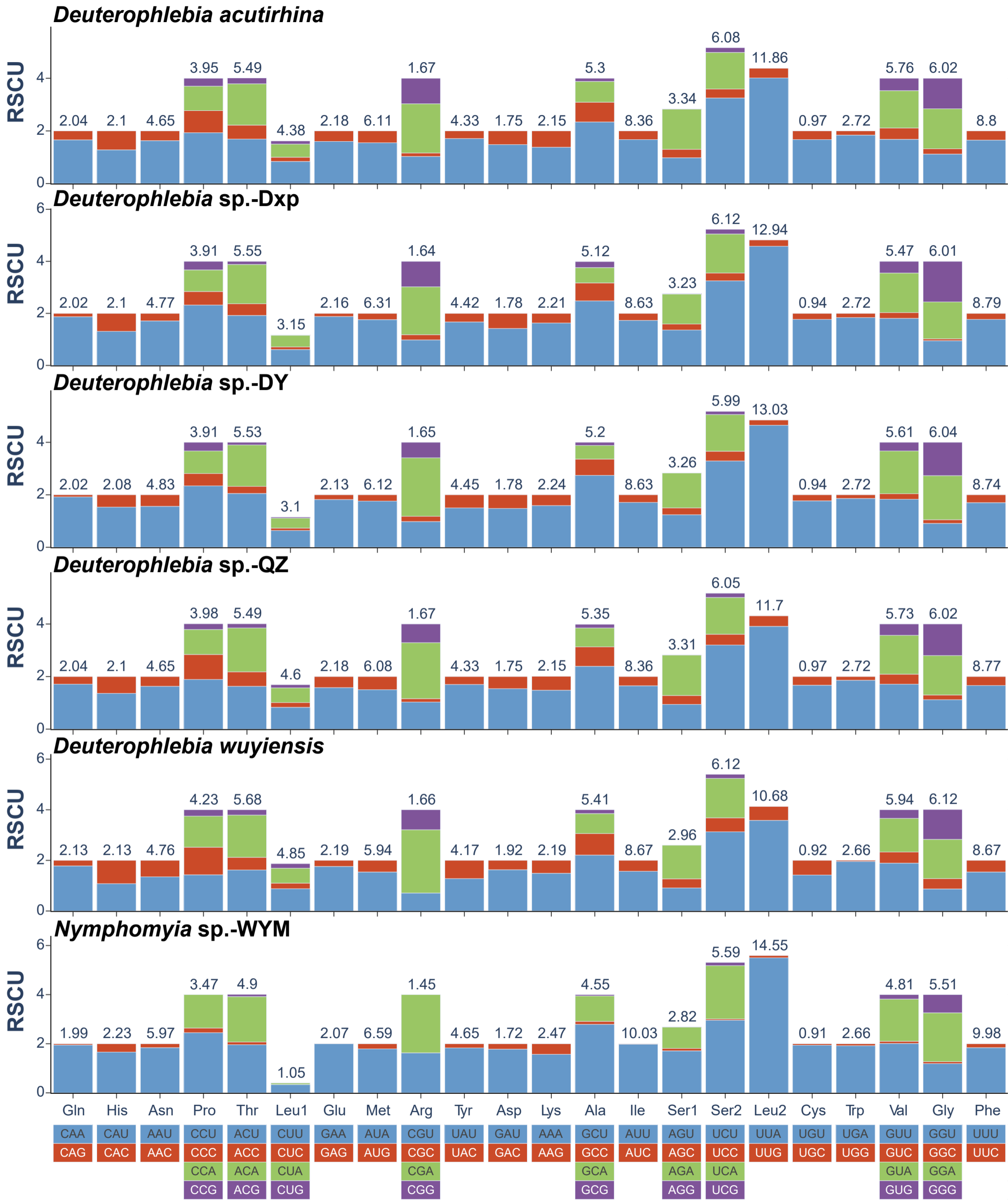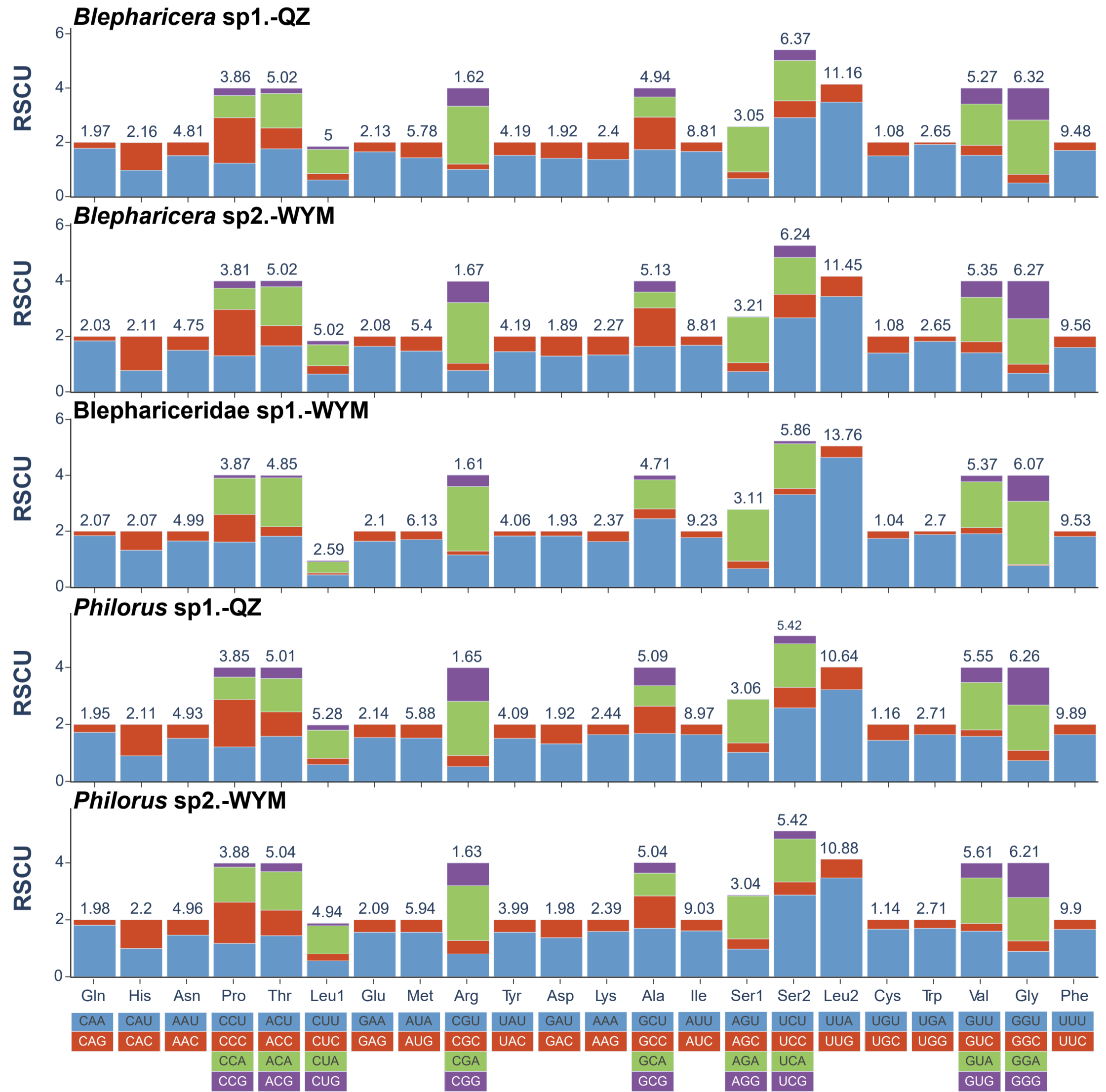

Supplement: Supplementary file 1 [file ijms-26-05714-s001.zip › Figure S1.pdf]

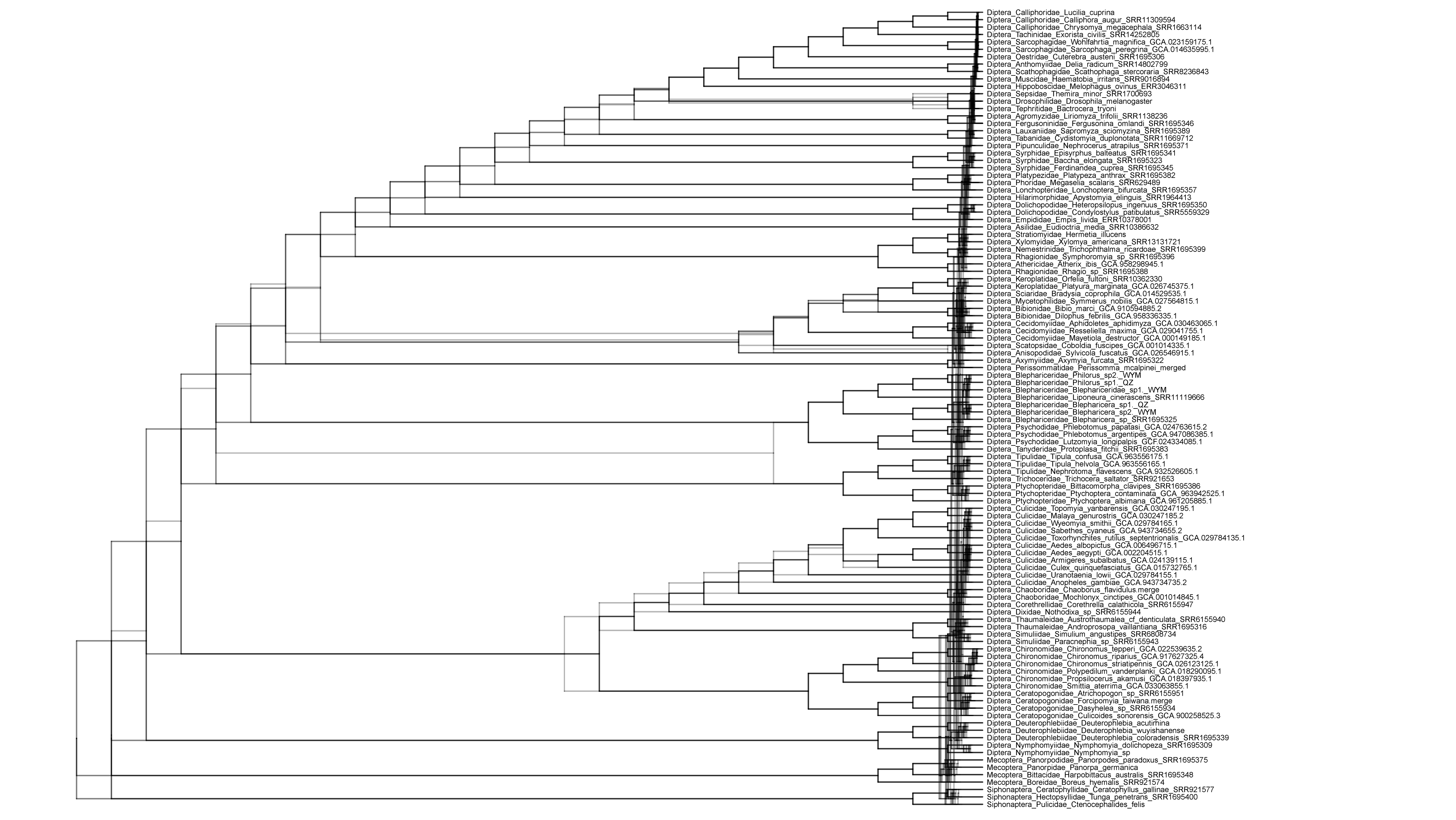

Supplement: Supplementary file 1 [file ijms-26-05714-s001.zip › Figure S2.jpg]

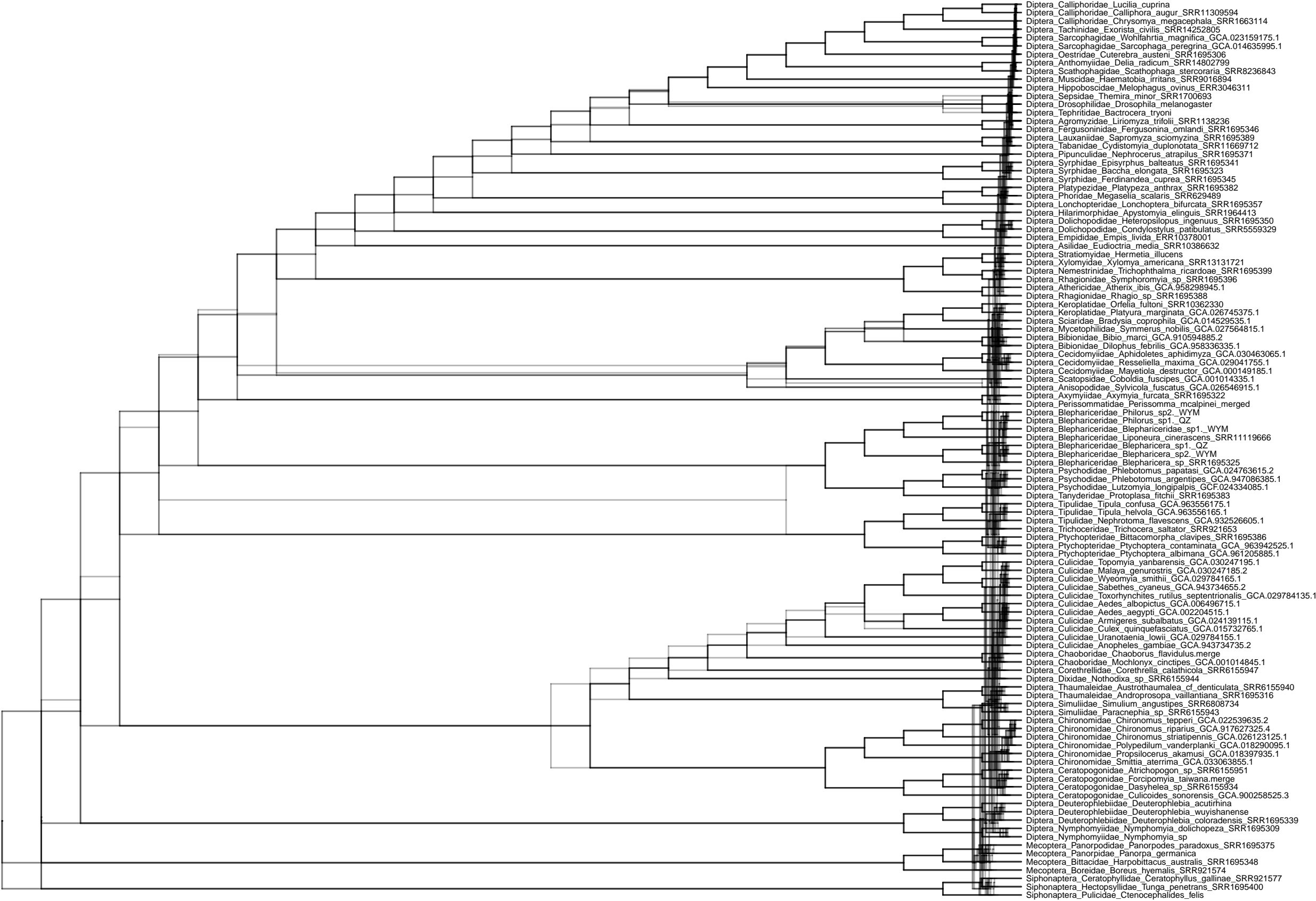

Supplement: Supplementary file 1 [file ijms-26-05714-s001.zip › Figure S2.pdf]

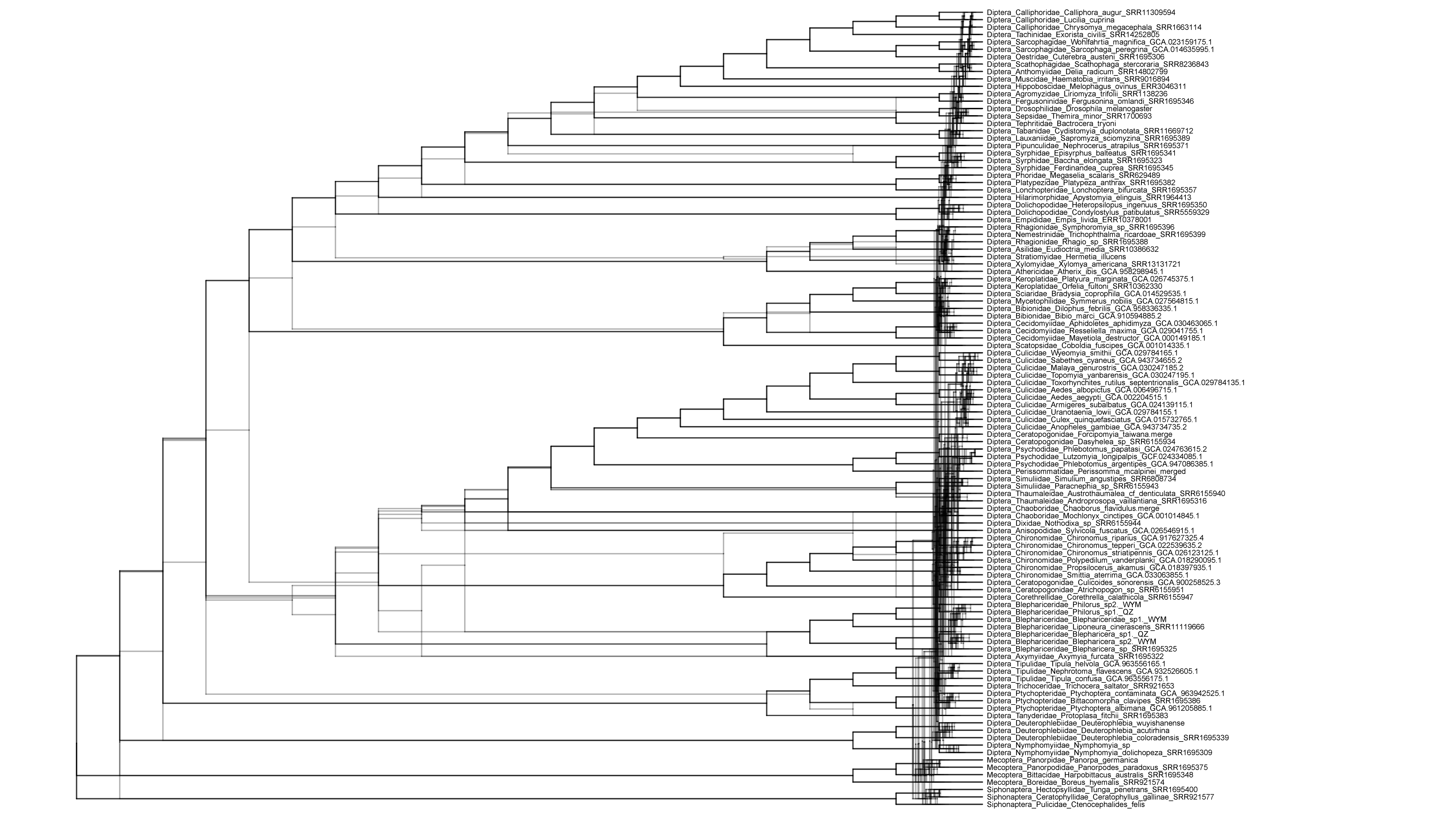

Supplement: Supplementary file 1 [file ijms-26-05714-s001.zip › Figure S3.jpg]

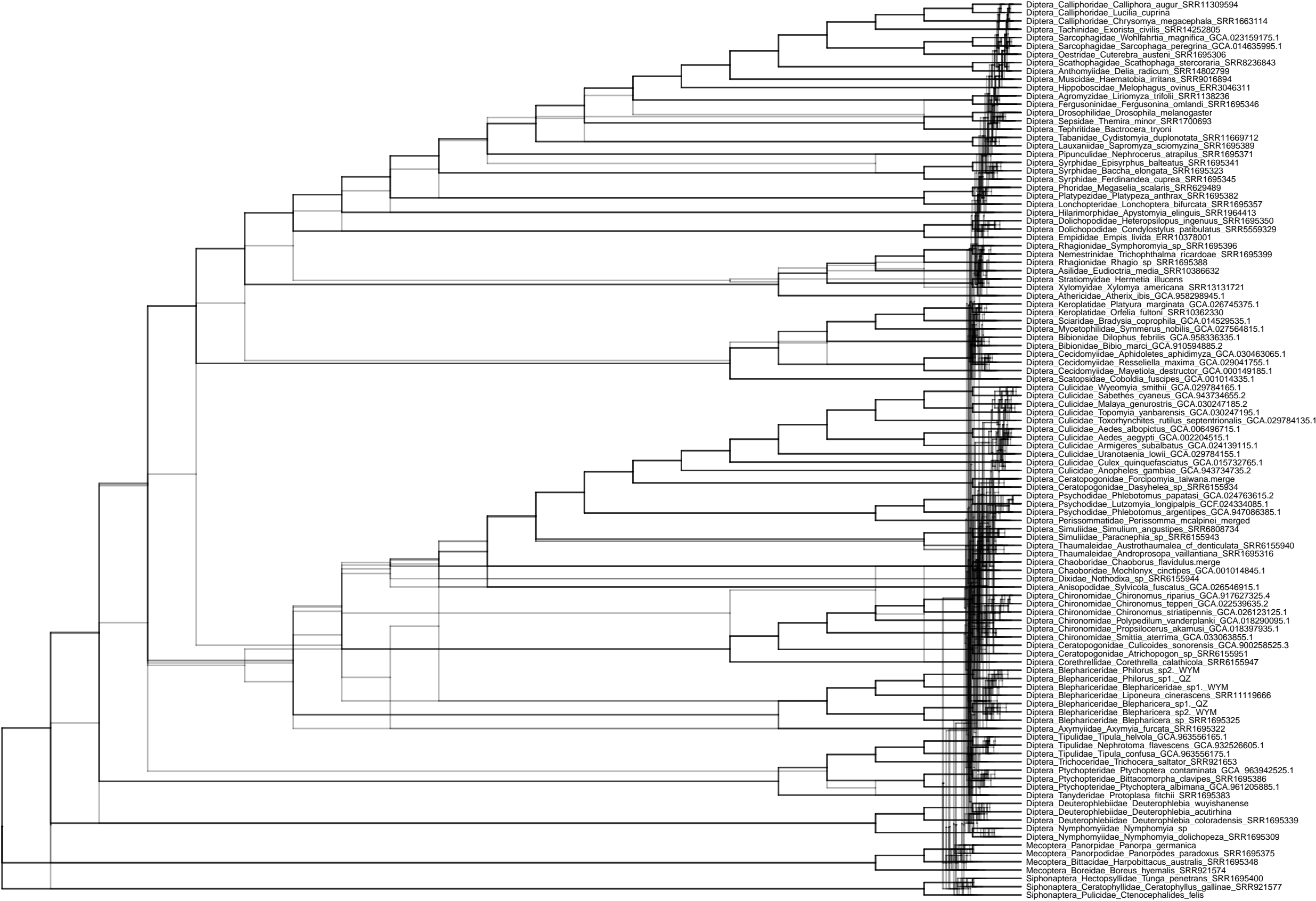

Supplement: Supplementary file 1 [file ijms-26-05714-s001.zip › Figure S3.pdf]

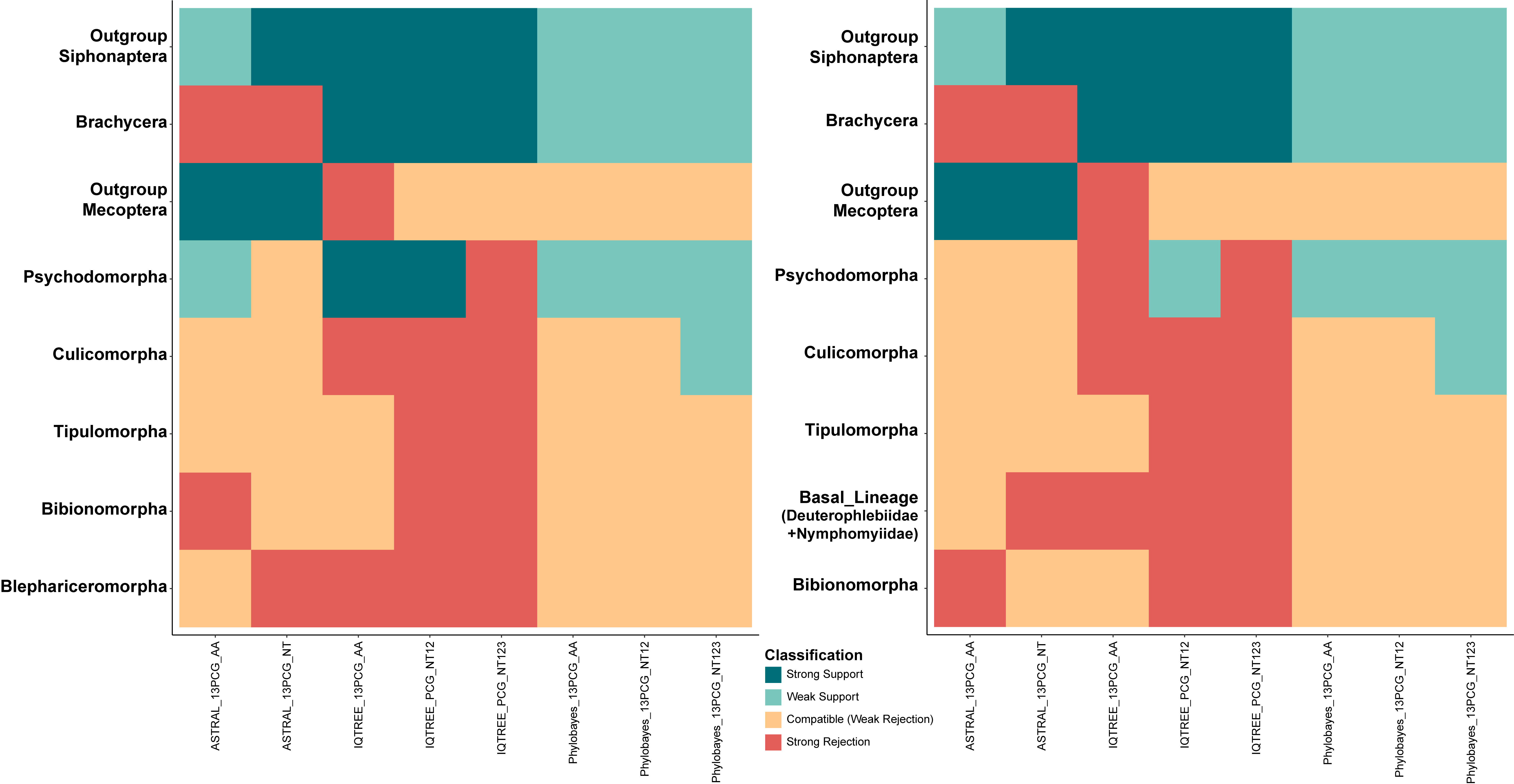

Supplement: Supplementary file 1 [file ijms-26-05714-s001.zip › Figure S4.jpg]

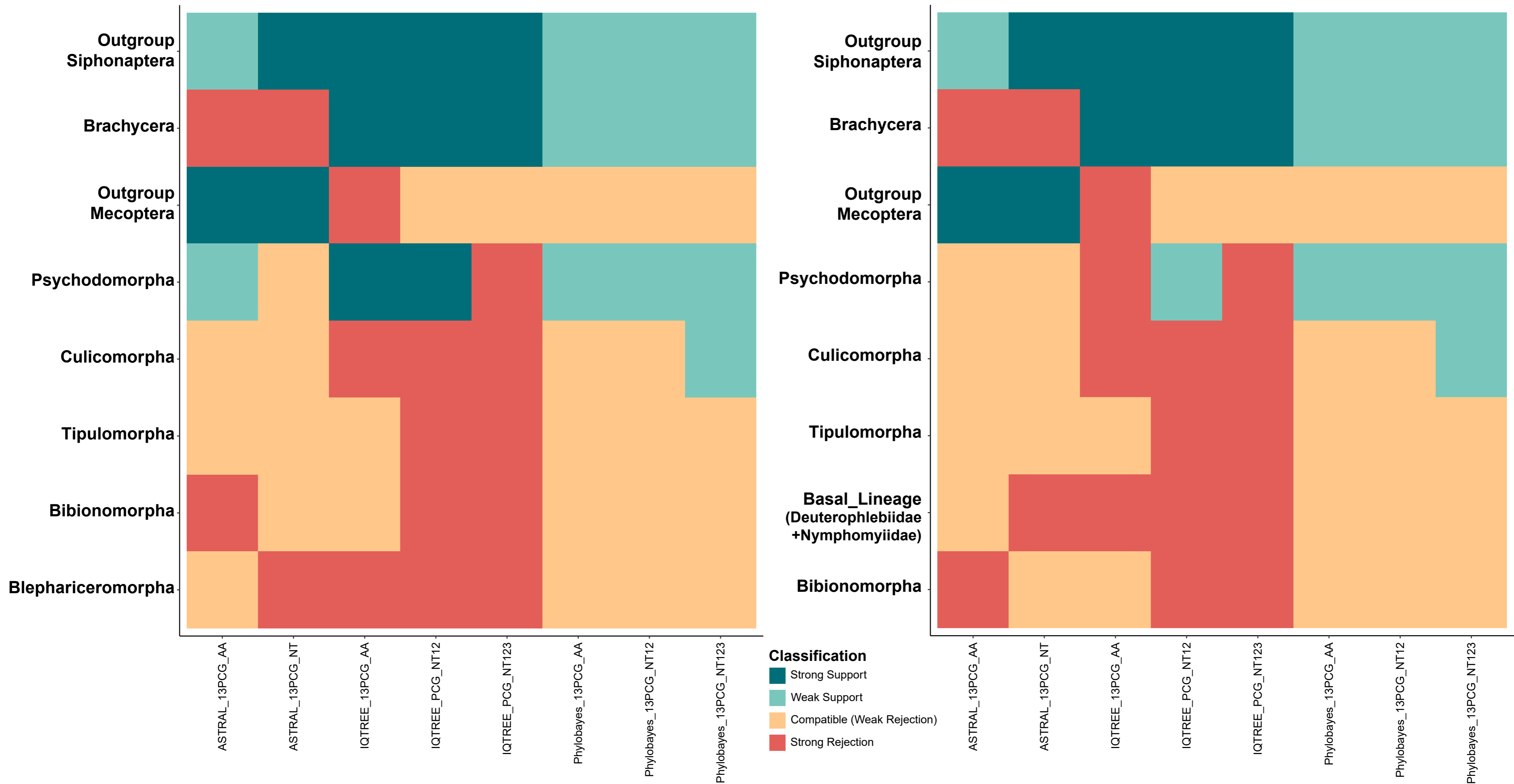

Supplement: Supplementary file 1 [file ijms-26-05714-s001.zip › Figure S4.pdf]
